# The evolution of the code during review: an investigation on review changes

**DOI:** 10.1007/s10664-022-10205-7

**Published:** 2022-09-20

**Authors:** Enrico Fregnan, Fernando Petrulio, Alberto Bacchelli

**Affiliations:** University of Zurich, Zurich, Switzerland

**Keywords:** Modern Code Review, Software analytics, Empirical software engineering

## Abstract

Code review is a software engineering practice in which reviewers manually inspect the code written by a fellow developer and propose any change that is deemed necessary or useful. The main goal of code review is to improve the quality of the code under review. Despite the widespread use of code review, only a few studies focused on the investigation of its outcomes, for example, investigating the code changes that happen to the code under review. The goal of this paper is to expand our knowledge on the outcome of code review while re-evaluating results from previous work. To this aim, we analyze changes that happened during the review process, which we define as *review changes*. Considering three popular open-source software projects, we investigate the types of review changes (based on existing taxonomies) and what triggers them; also, we study which code factors in a code review are most related to the number of review changes. Our results show that the majority of changes relate to evolvability concerns, with a strong prevalence of documentation and structure changes at type-level. Furthermore, differently from past work, we found that the majority of review changes are not triggered by reviewers’ comments. Finally, we find that the number of review changes in a code review is related to the size of the initial patch as well as the new lines of code that it adds. However, other factors, such as lines deleted or the author of the review patchset, do not always show an empirically supported relationship with the number of changes.

## Introduction

Code review is a widely adopted practice in software development in which one or more developers inspect the code written by another developer (Fagan 1976; Baum et al. 2019; Spadini et al. 2019) and recommend any useful or necessary changes to such code.

Code review was originally introduced as a formal process, involving a strictly regulated procedure (Weinberg and Freedman 1984; Yourdon 1989; Freedman and Weinberg 2000), e.g., Fagan’s inspection (1976). However, to better fit the needs of different developers’ teams and companies, code review has evolved towards less strict paradigms and more informal practices (Rigby et al. 2012). This lightweight process is often referred to as *Modern Code Review* (MCR). Nowadays, MCR has become the most popular approach adopted in practice to perform code review (Rigby and Bird (2013) 2013; Baum et al. (2016) 2016, 2017), often involving support from specialized software tools (Baum and Schneider 2016).

The main reported goal of code review (Bacchelli and Bird 2013) is to find defects and improve the overall quality of the code under review. However, previous studies (Bacchelli and Bird 2013; Czerwonka et al. 2015) reported the existence of a significant mismatch between what developers and managers expect from code review (for instance, find high-severity bugs) and what the real outcomes of this process are (e.g., finding a majority of low-level defects). The existence of such a mismatch was also confirmed by studies that analyzed the kind of changes happening in the code review process (Beller et al. 2014; Mäntylä and Lassenius 2009; Siy and Votta 2001): They highlighted how the majority of them do not fix major functional defects in the code, but instead focus on maintainability issues.

These results show the existence of a gap between what we *think to know* about code review and, on the contrary, what really happens in this process. For this reason, in this study our aim is to further expand the knowledge on the outcomes of code review. A sound understanding of the output of code review is fundamental to support, evaluate, and improve the code review process. In particular, the analysis of what kinds of code changes are triggered during code review, as well as which factors have an impact on the number of such changes, is important to inform the development of code review tools. Further insights in the code review process help project owners to evaluate their whole development process (e.g., do the caught defects match the expectations?).

**Fig. 1:**
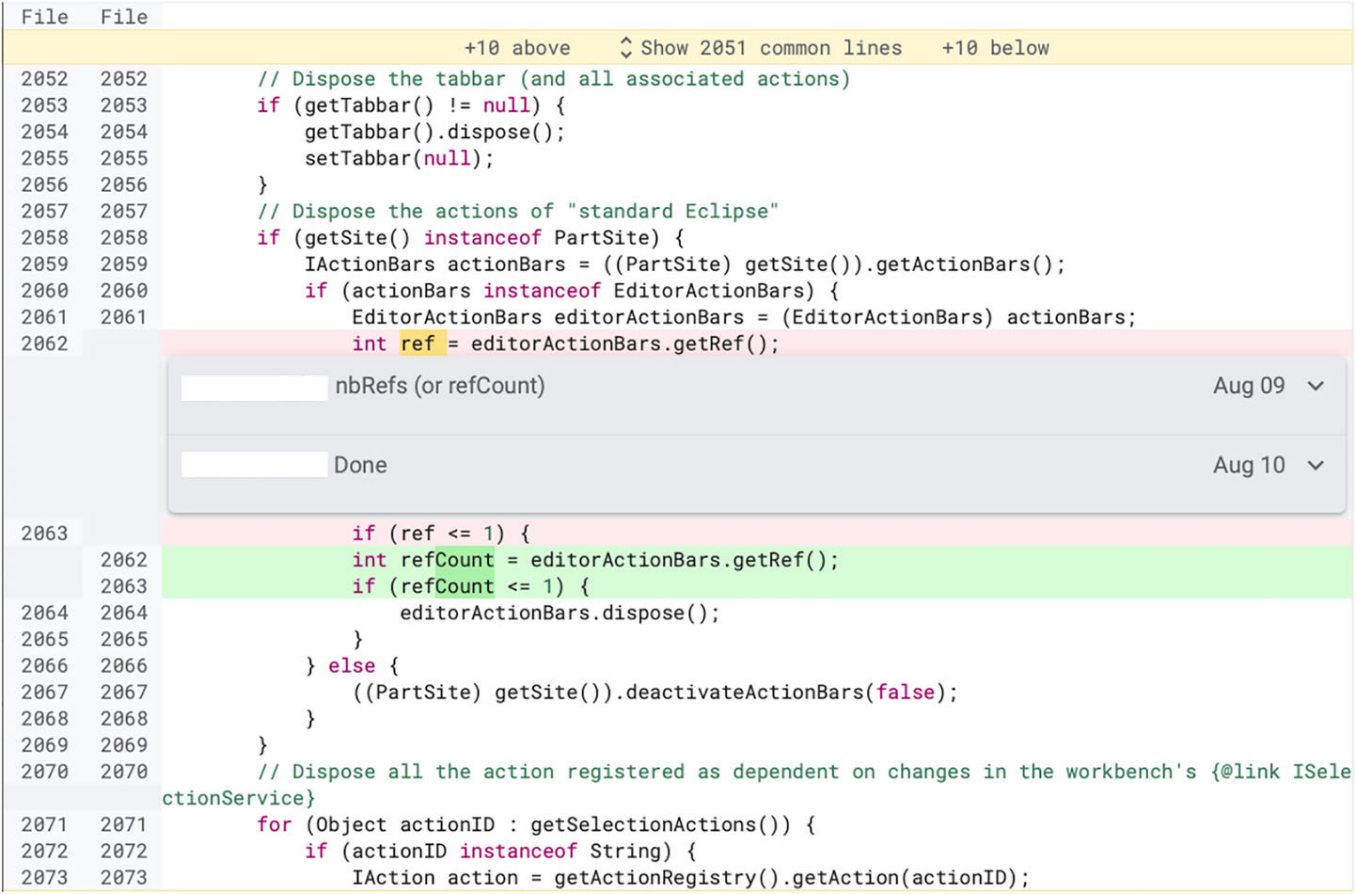
Example of review change (extracted from the online Gerrit repository of JGit). A reviewer asks for a variable to be renamed. In the following patch-set, the author of the change modifies the variable name and replies to the reviewer’s comment, marking the change as “done”. The complete review from which this file was extracted is available online (JGit 2021)

We define **review change** as every change done on the code under review during a modern code review (an example is shown in Fig. 1). In this paper, we present an empirical study that we conducted on review changes. First, we re-evaluate the findings by Mäntylä and Lassenius (2009) as well as Beller et al. (2014). Our aim is to revisit their study eight years after and challenge their findings on a different set of projects to update and expand our knowledge of the real outcomes of the code review process. To do so, we consider three projects (i.e., Android, JGit, and Java-client) different from the ones used in previous studies, which use Gerrit, a web-based open-source code review tool (Hamasaki et al. 2013). Our aim is to expand on the findings of Beller et al. (2014), which relied on only two projects, one of them with only 216 changes. JGit and Java-client are java-based projects, while Android contains different programming languages. We create a dataset containing 1,510 classified changes. We manually classify each of these changes according to the taxonomy used in the study by Beller et al. (2014) and Mäntylä and Lassenius (2009). Our classification is organized in three different granularity levels: category (i.e., *functional* or *evolvability*), type (e.g., *documentation* or *logic*), and sub-type (e.g., *textual*).

**Fig. 2:**
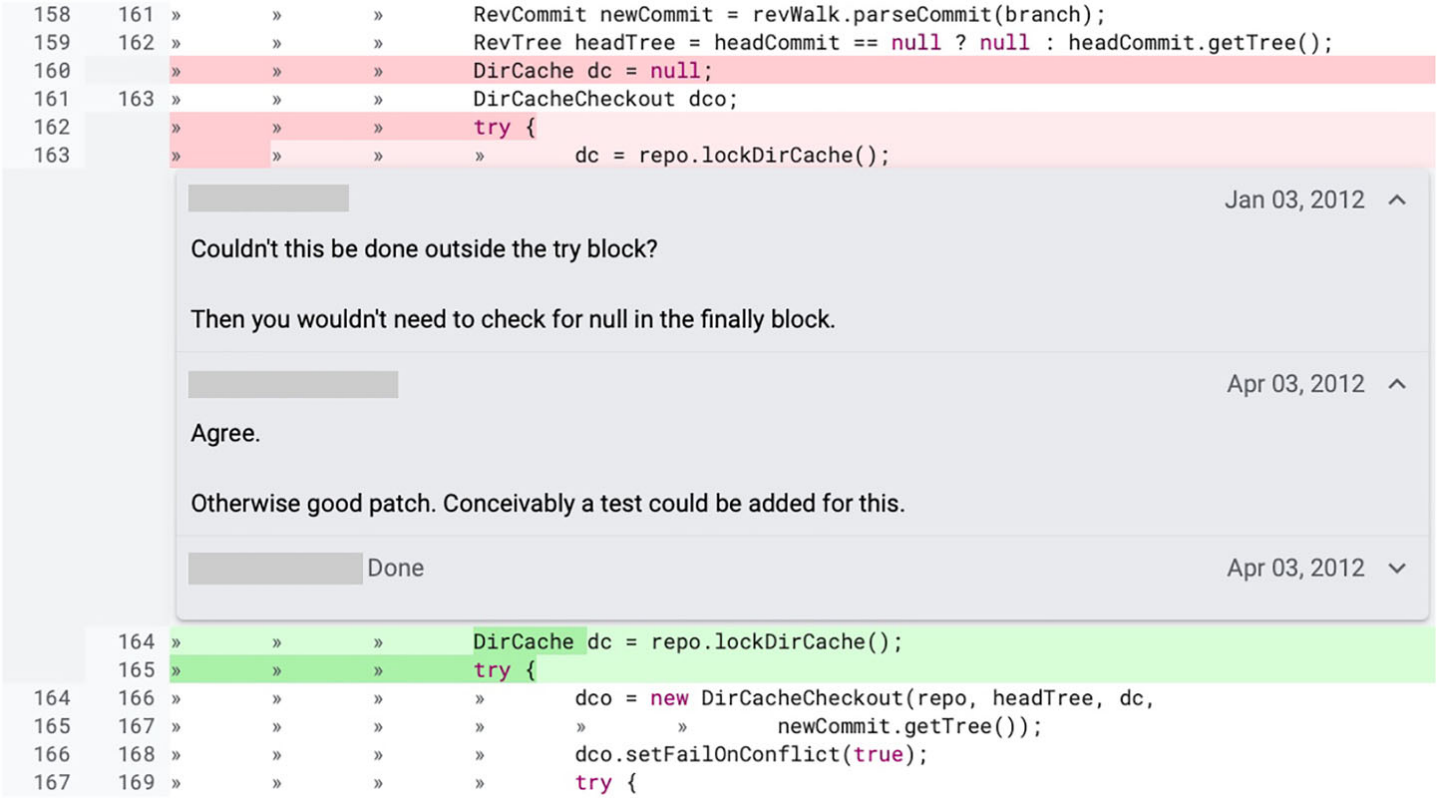
Example of *triggered by comment* review change (extracted from the online Gerrit repository of JGit https://git.eclipse.org/r/c/jgit/jgit/+/4869/1..2/org.eclipse.jgit/src/org/eclipse/jgit/api/CheckoutCommand.java). A reviewer asks for a statement to be moved outside of try-catch block. In the following patch-set, the author of the change modifies the variable name and replies to the reviewer’s comment, marking the change as “done”

**Fig. 3:**
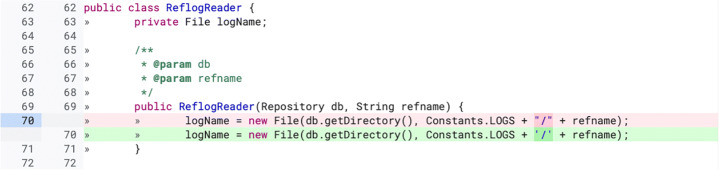
Example of *undocumented* review change (extracted from the online Gerrit repository of JGit https://git.eclipse.org/r/c/jgit/jgit/+/4747/2..3/org.eclipse.jgit/src/org/eclipse/jgit/storage/file/ReflogReader.java). In this change “/” is replace with the character ‘/’ inside a new File method

Second, we investigate what triggered a change: Was it a reviewer’s comment or something undocumented (e.g., changes triggered by a decision of the author himself or face to face discussions with the reviewers, as reported by previous studies (Beller et al. 2014; Bacchelli and Bird 2013))? Knowing the causes for a change increases our understanding of how developers act in code review. Furthermore, we investigate whether certain types of changes have a stronger correlation with one specific cause. Our hypothesis is that some low-complexity defects (e.g., naming issues) might be fixed by the author in a later patch without any input from reviewers, while hard to notice defects (e.g., functional bugs) might require the reviewers’ intervention. Based on our results, we analyze the ratio of triggered and undocumented changes contained in a revision. ‘Triggered by a comment’ changes are caused by a reviewer’s comment (as shown in Fig. 2), while ‘undocumented’ changes are not caused by a reason clearly recognizable in the developers’ interactions in Gerrit (an example is shown in Fig. 3).

Subsequently, we further extend the findings of previous studies, by building logistic regression models to evaluate which factors might impact the ratio of triggered by a comment and undocumented changes in a revision. Our aim is to collect additional insight on the presence of special characteristics in a review that might explain, for instance, a higher number of undocumented changes.

Finally, we study which features are correlated to the number of changes that take place in a review. Understanding the characteristics of a code change under review that are likely to require more changes before being accepted can support practices, such as help allocate more time or reviewers to change-prone pull requests. We extract a set of features from Gerrit repositories of the projects contained in our dataset and create a Generalized Linear Model (GLM) (Fahrmeir et al. 2013) for each project and one for the whole dataset to measure such a correlation. In this investigation, we expand on the work of Beller et al. (2014) by integrating new features in the original set considered by the authors and applying our analysis to all three projects in our dataset as well as to their combination to test if our assumptions are project-independent. Moreover, to further advance the findings of previous work, we investigate two new projects (EGit and Platform UI) from the CROP (Paixao et al. 2018) dataset. In this step, we consider new possible features that might impact the complexity of a review: e.g., the length of the description of a patch-set or the presence of bots in a review. We build GLMs models to investigate the correlation of this new set of variables to the number of changes in a review.

Concerning the distribution of code review changes in the taxonomy, our study confirms that previous findings (Beller et al. 2014; Mäntylä and Lassenius 2009) are also valid on the three newly considered software systems. That is, the majority of the changes concern the evolvability aspects of the code under review, with documentation and organization changes being the most preponderant. Regarding the proportion of commented and undocumented changes, we observe an opposite trend: the majority of the changes in our dataset are undocumented. Finally, our Generalized Linear Models revealed how features like size or lines of code added in the initial patch to review are statistically significant factors to predict the number of changes. Other features’ impact differs based on the project.

The contributions of this paper are the following:

**A dataset of 1,510 classified review changes:** We manually classified review changes according to the taxonomy developed by previous studies (Beller et al. 2014; Mäntylä and Lassenius 2009). We define a review change as a group of continuous modified code lines (code chunk) or multiple logically related code chunks (see Section 3.3). The dataset contains 1,510 changes from three open-source projects (i.e., Android, JGit, and Java-client) that use Gerrit as tool to perform code reviews. Furthermore, each change is labeled based on the reason behind it: ‘Triggered by a comment’ or ‘Undocumented’. Android contains a variety of programming languages, while JGit and Java-client are java-based projects. Similarly to previous studies (Gousios et al. 2016; Golzadeh et al. 2021; Pascarella et al. 2019), we reported the dataset as a contribution as it can be beneficial for future research. Our dataset is publicly available in our replication package to allow researchers to use it or build upon it in future studies.
**Empirical data on the categories, types, and subtypes of review changes:** Most review changes (approximately 90%) involves only maintainability issues, while the remaining impact functional aspects of the code. In the former category, the majority of changes is related to the documentation and the structure of the code, while the latter is mainly formed by changes impacting logic and function calls. These results (1) corroborate the finding of previous studies (Beller et al. 2014; Mäntylä and Lassenius 2009) and (2) advance our understanding of the outcomes of the code review process by considering a finer level of classification (sub-types), not considered in the study of Beller et al. (2014), on a larger set of reviews from three active open-source projects (as opposed to fewer industrial and students’ reviews, as done previously (Mäntylä and Lassenius 2009)).
**Empirical data on the reason behind review changes:** Our results report that the majority of changes are not caused by a comment, but their percentage varies greatly based on the project: 64.6% in Android, 60.7% in JGit, and 80.7% in Java-client. Furthermore, we found that no statistically significant correlation exists between the cause of a change and its category or type for the three separate projects. However, considering all projects, we identified a weak correlation between the cause of a change and its type.
**A correlation analysis of the number of changes and their features:** We applied four Generalized Linear Models (GLMs), one for each of three project in our dataset and one that takes into account all projects, to analyze the correlation between the number of changes in a review and its characteristics. We found that metrics related to the size of a review are significant factors to predict the number of changes. Furthermore, informed by the results of this initial investigation, we expanded our analysis on a larger set of metrics and projects.

### Structure of the Paper

Section 2 presents related work and background studies on which our investigation is based. Section 3 contains our research questions and explains how the dataset used in our investigation was constructed. Sections 4.1, 4.2 and 4.3 illustrate the methodology and findings of each research question. Section 5 discusses the results and implications of this work. Finally, Section 6 presents the threats to the validity of our investigation and Section 7 concludes this study.

## Background and Related Work

In this section, we explain the background concepts on code review and present the literature related to the goals of our paper.

### Terminology

In the context of this paper, we refer to specific concepts that we define here:

**Review:** A review is the whole process in which reviewers inspect a developer’s contribution before allowing it to be merged in the project’s codebase. In a review, there are typically multiple reviewers Rigby et al. (2012) and Sadowski et al. (2018), who look for defects in the code, ask for improvements, and evaluate the contribution. On Gerrit, a review can be formed by multiple rounds called *revisions* and organized in patches. A reviewer can ask the author of the code for changes and they will address them in a follow-up patch.
**Revision:** A review comprises one *revision* or more, each one uploading a single patch (i.e., a set of code changes to possibly multiple files). The first revision uploads the patch that the author sends for review in the first instance. The following (optional) revisions upload additional patches that the author has decided to submit to further modify the code, usually to address reviewers’ concerns. Each patch is subject to the reviewers’ scrutiny.
**Review change:** As reviewers inspect a patch and discuss it with the author, they may recommend modifications to the submitted patch. Also, an author may realize a mistake after having submitted a patch. A *review change* is a change applied to the code under review and uploaded in a patch of a subsequent revision.

### Code Review

Code review was originally born as a formal, highly structured approach based on physical meetings. Among the different methods proposed, Fagan’s inspections (1976) are considered to be the first formal procedure for code reviews. However, this form of code inspections had multiple shortcomings, the most relevant being the synchronous nature of the process with a high number of meetings. Numerous studies have shown that these meetings are costly and do not significantly increase the quantity of defects found in the code (Johnson and Tjahjono 1998; Bianchi et al. 2001).

To address the inefficiencies of code inspections, the review practice shifted towards a new paradigm, often called Modern Code Review (MCR) (Rigby and Bird 2013) (other names are *change-based review* (Baum and Schneider 2016), *differential code review* (Bernhart and Grechenig 2013), and *patch review* (Baum et al. 2016)). MCR is a lightweight, informal approach focused on reviewing code changes and characterized by less strict practices also involving support from software tools (Rigby et al. 2012).

The purpose of these software tools is to support the logistics of the code review process by allowing developers to inspect and discuss code changes before merging it in the project code-base (Baum and Schneider 2016). Researchers proposed improvements to these tools creating prototypes that further support developers during code review: e.g., organizing or decomposing files using factors as the type of change or the semantic context (Wiggins et al. 2019; Rachamadugu et al. 2008; Barnett et al. 2015; McGraw 2008; Zhang et al. 2015). Some large companies have also developed their own tools to tailor the review process to their needs: e.g., Microsoft’s CodeFlow (Czerwonka et al. 2018), Google’s CRITIQUE (Sadowski et al. 2018), or Facebook’s Phabricator (Feitelson et al. 2013).

**Fig. 4:**
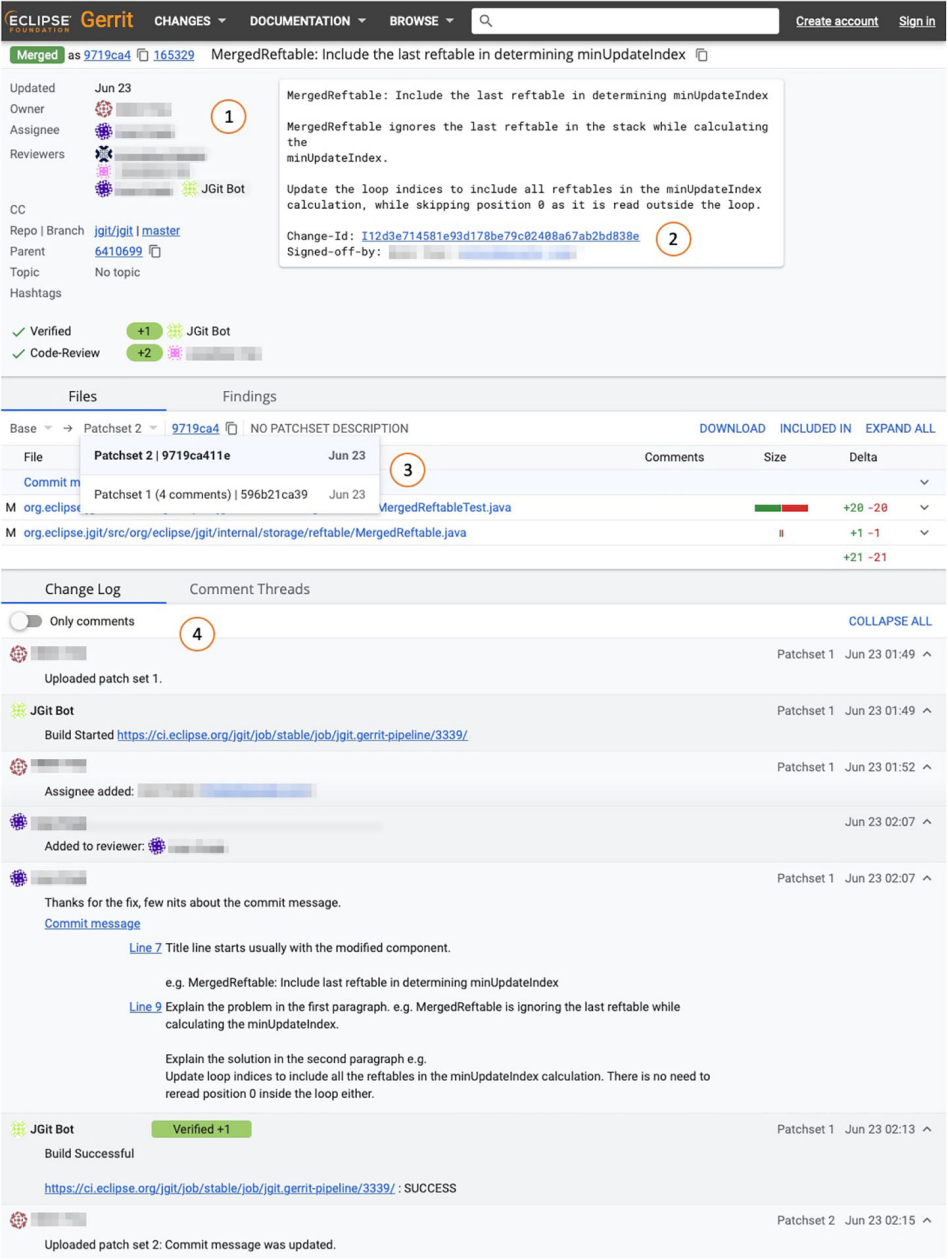
Example of code review as displayed in Gerrit

In our study, we focus on Gerrit, an open-source, web-based code review tool initially developed by Google (Gerrit 2015). Figure 4 shows a code review (extracted from the *Eclipse JGit* project) done with Gerrit. Gerrit provides a list of the developers (including bots, if any) involved in the review process (part in Fig. 4): the author of the change under review, the assignee, and the reviewers. It also displays further information on the change under review: e.g., the repository and the branch to which it belongs. Gerrit assigns a unique ID to the change under review and allows the owner to add a description of the code under review (part in Fig. 4). Part of the tool reports information on the files contained in each patch uploaded for review, as well as displaying the number of patches uploaded. The drop-down menu shows the patchsets uploaded during the review, together with the unique ID assigned to the patch-set and the number of reviewers’ comments added. Clicking on a file, a reviewer can visualize the *diff* between the version of the file in the patch currently selected and the version of the file contained in a previous patch. Finally, part shows the log of the activities for the review. Gerrit keeps track of every time a new patch is uploaded or a reviewer is added. Furthermore, reviewers’ comments are also displayed here (as shown in Fig. 4).

**Fig. 5:**
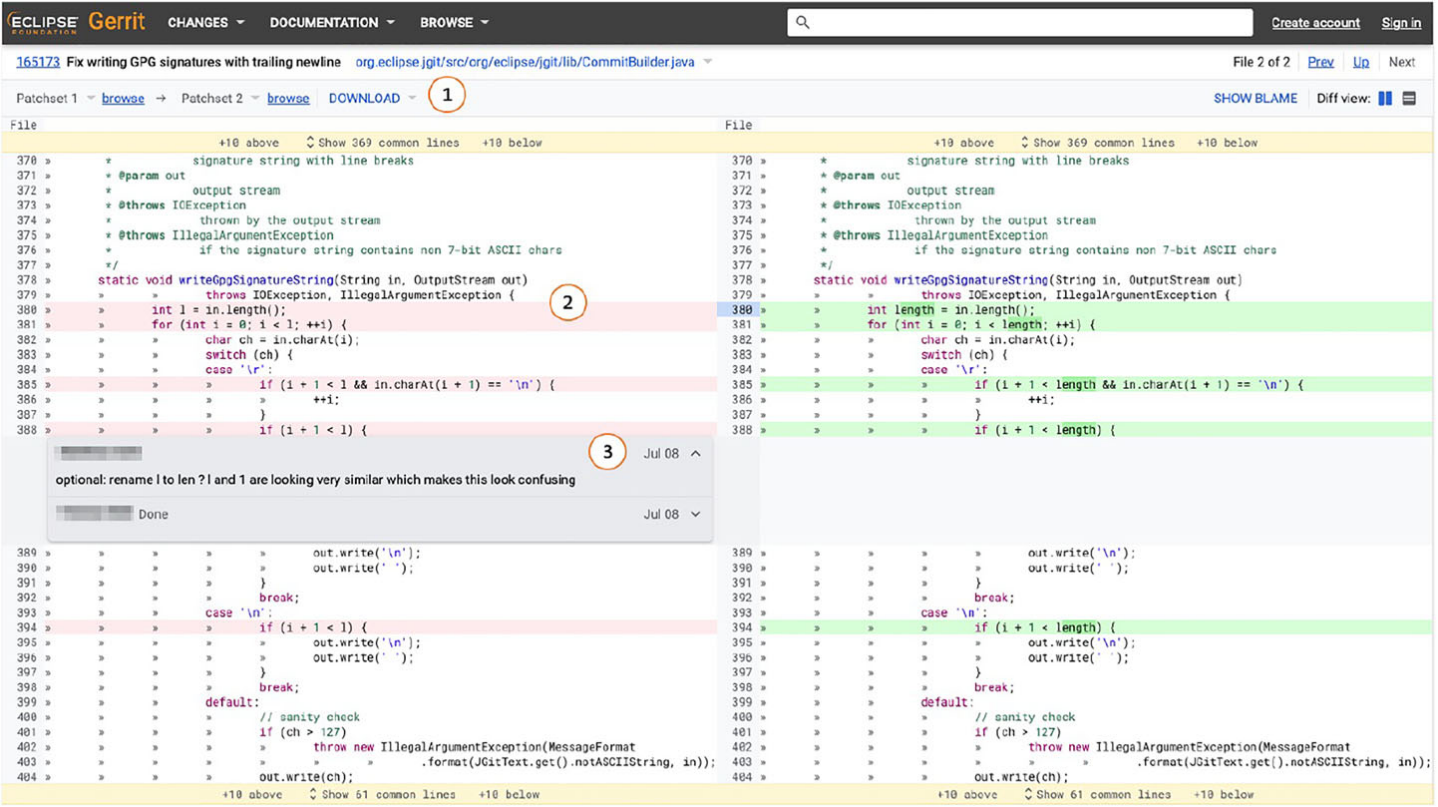
Example of the file diff for review as displayed in Gerrit. The complete review from which this file was extracted is available online (https://git.eclipse.org/r/c/jgit/jgit/+/165173/1..2/org.eclipse.jgit/src/org/eclipse/jgit/lib/CommitBuilder.java, July 2020.)

Figure 5 shows the diff of a file as displayed for review in Gerrit. Part shows information about the selected file: its name, the review title, and the patchsets between which the diff is displayed. Gerrit highlights the code chunks that changed between the two patchsets (an example is shown in part of Fig. 5). Finally, part shows an in-line comment left by a reviewer proposing to rename the variable *l* to *len* (then, the author decided to further change the variable name in *length*). Gerrit allows adding further comments to an initial comment left by a reviewer, starting a discussion. In this example, the owner of the patchsets under review replied marking the reviewer’s suggestion as *done*.

We focus on Gerrit due to its vast diffusion among open-source software projects (McIntosh et al. 2016; Paixao et al. 2018) and the possibility to extract information on the review process through its openly accessible API. The review process in Gerrit is organized in patch sets containing the files to be reviewed. After the first patch is uploaded and examined by the reviewers, the author of the modification can upload further patches to implement the changes asked by and discussed with the reviewers through a commenting feature offered by the tool (Mukadam et al. 2013). This process continues until the code is deemed ready to be merged in the code-base or rejected.

### Classifying Review Changes: Taxonomies of Code Changes

Previous research focused on classifying software defects, leading to the creation of multiple taxonomies (e.g., Chillarege et al. (1992) 1992 and Siy and Votta (2001) 2001). Mäntylä and Lassenius (2009) surveyed existing defects taxonomies and addressed their shortcomings creating a new more comprehensive taxonomy of code defects. This taxonomy contains three main categories: evolvability defects, functional defects, and false positives. The evolvability and functional categories were further divided into multiple sub-categories.

**Fig. 6:**
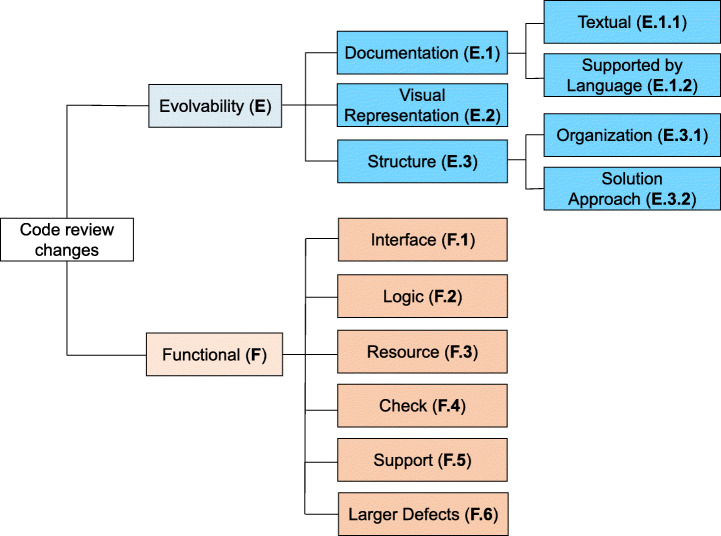
Taxonomy of the changes in code review

Beller et al. (2014) emphasized the human aspects of code review and suggested to use the taxonomy by Mäntylä and Lassenius (2009) to classify review changes, substituting the terms ‘defects’ or ‘fix’ with ‘change’ (Beller et al. 2014). This substitution is to account for the discretionary character of the code review process, in which “improvements” recommended by some reviewers might not be considered as such by a different group of reviewers (Beller et al. 2014). Beller et al. (2014) further refined the classification by Mäntylä and Lassenius (2009), eventually producing the taxonomy shown in Fig. 6.

The taxonomy in Fig. 6 has two main categories: evolvability changes and functional changes. The former includes changes related to the maintainability of a software system (e.g., changes aimed at improving the readability of the code), while the latter contains changes that impact the functionality of the system. Each category has been further divided in subgroups (or types).

Moreover, all these types can be further refined into subtypes to convey an even finer level of information. This new level of the taxonomy includes more than 40 classes. While describing the change types, we briefly presented the most relevant subtypes per each type. A complete description of each subtype can be found in the online appendix, together with a more detailed description of each type of review change.^1^

**Fig. 7:**
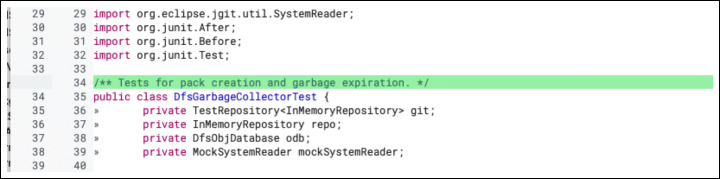
Example of ‘Textual - Comment’ change: A comment line has been added to explain the purpose of the following tests. The real-world code review in which this change took place is available online (https://git.eclipse.org/r/c/jgit/jgit/+/99371/3..4/org.eclipse.jgit.test/tst/org/eclipse/jgit/internal/storage/dfs/DfsGarbageCollectorTest.javahttps://git.eclipse.org/r/c/jgit/jgit/+/99371/3..4/org.eclipse.jgit.test/tst/org/eclipse/jgit/internal/storage/dfs/DfsGarbageCollectorTest.java, December 2017)

**Fig. 8:**
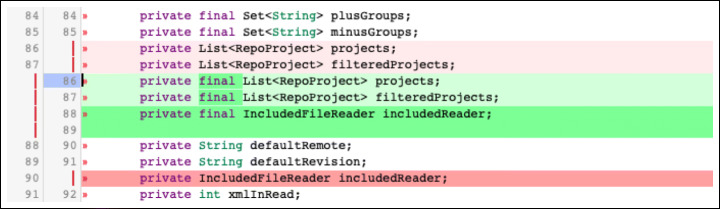
Example of ‘Supported by language - Immutable’ change: The Java *final* keyword has been added to the variables. The real-world code review in which this change took place is available online (https://git.eclipse.org/r/#/c/48522/1..2/org.eclipse.jgit/src/org/eclipse/jgit/gitrepo/ManifestParser.java, May 2015.)

**Fig. 9:**
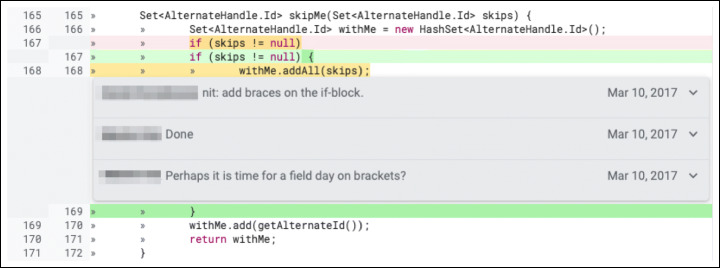
Example of ‘Visual representation - Bracket usage’ change: Missing braces have been added in the *if* statement to improve the code style. The real-world code review in which this change took place is available online (https://git.eclipse.org/r/c/jgit/jgit/+/55148/6..7/org.eclipse.jgit/src/org/eclipse/jgit/internal/storage/file/CachedObjectDirectory.java, April 2017.)

**Fig. 10:**
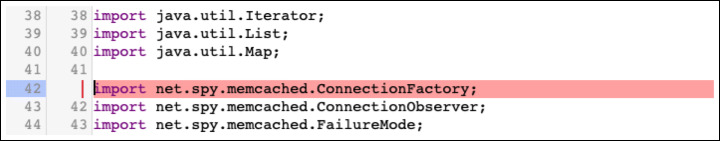
Example of Organization - Dead Code change: An unused import statement has been removed from the code. The real-world code review in which this change took place is available online (http://review.couchbase.org/#/c/14574/1..2/src/main/java/com/couchbase/client/CouchbaseConnection.java, April 2012.)

**Fig. 11:**
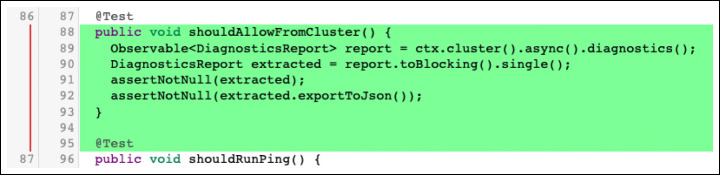
Example of Solution approach - New functionality change: A new test has been added in a test class. The real-world code review in which this change took place is available online (http://review.couchbase.org/#/c/100690/1..2/src/integration/java/com/couchbase/client/java/DiagnosticsTest.java, November 2018.)

**E. Evolvability Changes**: The evolvability category consists of three classes. We introduce them using examples from our dataset.
  **E.1 Documentation:** Changes that impact the information given in the code to facilitate its comprehension by humans. This group of changes include:
    **E.1.1 Textual changes:** Modifications in how information about the code is conveyed to other developers. The two most prominent subtypes of textual changes are ‘comment’ and ‘naming’. A ‘comment’ change (as seen in Fig. 7) often involves missing, wrong, or incomplete code documentation. A ‘naming’ change usually happens to fix unclear or inconsistent variables naming.
    **E.1.2 Supported by language:** Changes related to specific programming languages features used to convey information to humans: e.g., the *final* keyword in Java is reported to be used mainly for documentation purposes (Mäntylä and Lassenius 2009). Figure 8 shows an example, which belongs to the ‘immutable’ subtype. Another common subtype of supported by language changes is ‘element type’ changes, which occur when the type of a software element (e.g., a variable) is incorrect.
  **E.2 Visual representation:** These are changes that deal with the layout of the code: e.g., adding blank lines or adding/removing brackets to improve the code style (Fig. 9 shows an example of the latter). Other common subtypes of visual representation changes include splitting excessively long lines into multiple lines of code (‘long line’ changes) or fixing wrong indentation of the code (‘indentation’ changes).
  **E.3 Structure:** This category regards changes that modify the structure of the project and is divided in:
    **E.3.1 Organization changes:** Changes with the aim of reorganizing the project’s code. They often involve removing unused portions of code (‘dead code’ change subtype) or moving portions of the code to different packages or classes without modifying the functionality of the code (‘move functionality’ subtype). Figure 10 shows an example of ‘dead code’ change, taken from our dataset.
    **E.3.2 Solution approach changes:** changes that modify the way in which a solution is implemented in the code, without, however, modifying its functionality. Examples include old functions that needs to be updated (‘change function’ subtype) or removals of code that is executed without serving any meaningful purpose (‘semantic dead code’ subtype).
      Furthermore, among the solution approach changes, we find ‘new functionality’ changes. This subtype of changes includes new code added to make the software better maintainable in the long run. We include in this category the addition of new tests, as depicted in Fig. 11.

**Fig. 12:**
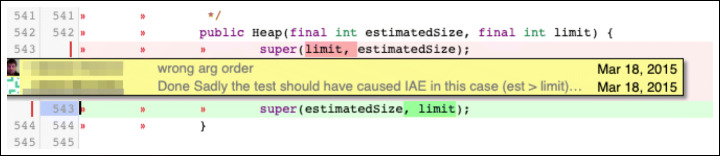
Example of ‘Interface - Function call’ change: Parameters are passed to the function in the wrong order. The real-world code review in which this change took place is available online (https://git.eclipse.org/r/#/c/44126/2..3/org.eclipse.jgit/src/org/eclipse/jgit/util/TemporaryBuffer.java, March 2015.)

**Fig. 13:**
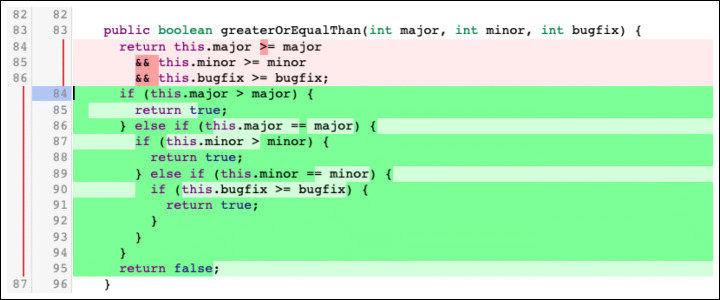
Example of ‘Logic - Computation’ change: The method logic has been changed to check variables separately in multiple conditional statements. The real-world code review in which this change took place is available online (http://review.couchbase.org/#/c/45557/1..2/src/test/java/com/couchbase/client/CbTestConfig.java, January 2015)

**Fig. 14:**
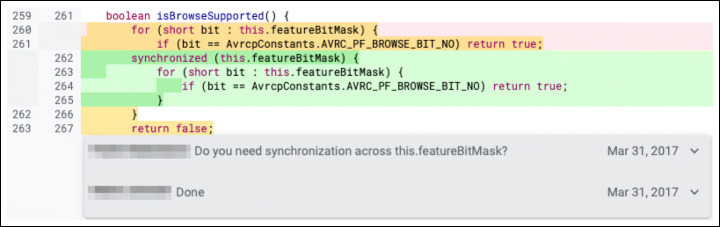
Example of ‘Resource - Data and Resource manipulation’ change: The variable *featureBitMask* has been protected against concurrent access using the java *synchronized* keyword. The real-world code review in which this change took place is available online (https://android-review.googlesource.com/c/platform/packages/apps/Bluetooth/+/358197/5..6/src/com/android/bluetooth/avrcp/AvrcpHelperClasses.java, April 2017.)

**Fig. 15:**
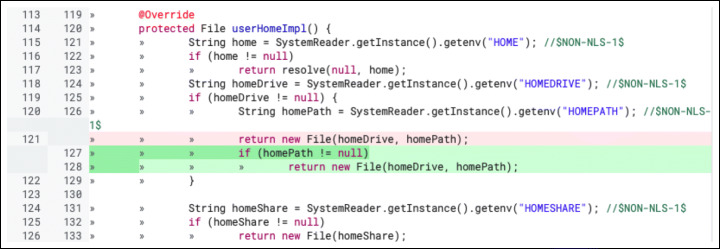
Example of ‘Check - Check variable’ change: The variable *homePath* needs to be non-null before it is passed to the constructor of *File*. The real-world code review in which this change took place is available online (https://git.eclipse.org/r/c/jgit/jgit/+/9549/1..2/org.eclipse.jgit/src/org/eclipse/jgit/util/FS_Win32.java, June 2013.)

**F. Functional changes**: Functional changes are divided in six types. We describe them, together with their most prominent subtypes and examples from our dataset.
  **F.1 Interface:** Changes that impact the interaction with other parts of the codebase. Calling wrong methods or passing incorrect parameters are common examples of subtypes in this class of changes. Figure 12 reports an example.
  **F.2 Logic:** Changes to logical operations in the code, such as comparison statements, computation mistakes, and optimization of the logic of inefficient algorithms. Figure 13 shows a change to a logic comparison.
  **F.3 Resource:** Changes that involve the handling of variables. They are divided into three major subtypes: (i) ‘variable initialization’ changes deal with variables left non-initialized before their use; (ii) ‘memory management’ changes fix issues in handling the memory system; (iii) ‘data and resource manipulation’ are changes that concern how data are manipulated or released. Figure 14 shows an example of the last subtype, where the Java synchronized instruction to handle concurrent access to a variable.
  **F.4 Check:** Changes that fix not-handled states issues. They typically imply the addition/modification of checks on a variable (‘check variable’ subtype, Fig. 15) or on the value returned by a function (‘check function’ subtype).
  **F.5 Support:** Changes related to a support system or library. For instance, using the wrong version of an external API might introduce defects in the code that need to be addressed.
  **F.6 Larger defects:** Major changes that require a significant effort and knowledge of the system. Usually, they span across multiple files or packages and fix missing or incorrect functionalities. These changes can be divided into three subtypes: (i) ‘completeness’ changes regard fixing features that are only partially implemented; (ii) ‘GUI’ changes modify inconsistencies in the User Interface; (iii) ‘check outside code’ changes are modifications that require to inspect parts of the code not included in the review patch set.

### Code Review Outcomes

Code review is a widely adopted software practice (Rigby and Bird 2013). However, the knowledge on the actual effects of code review and how code review effects can be automatically assessed is still limited. For instance, previous studies (Bacchelli and Bird 2013; Czerwonka et al. 2015) revealed the existence of a mismatch between what managers want to obtain from code review (e.g., detecting sever defects) and what the real outcomes of the code review process are (e.g., catching only a limited number of low level defects).

For this reason, a number of empirical studies investigated the actual outcomes of the code review process (Bacchelli and Bird 2013; Mäntylä and Lassenius 2009; Uchôa et al. 2020). For instance, the work of Morales et al. (2015) focused on the impact that modern code review has on software design quality (Morales et al. 2015). Looking at three open-source systems (Qt, VTK, and ITK), their investigation revealed how a higher review coverage has an effect on the presence of anti-patterns. This finding suggested a positive impact of code review in increasing the software quality of a project. Moreover, the authors reported that reviewers’ participation plays a key role in reducing the number of anti-patterns. Focusing on developers’ interactions, El Zanaty et al. (2018) investigated how often reviewers discuss design-related issues (El Zanaty et al. 2018). Their results provide evidence that design issues are rarely discussed during code review.

The study of Uchôa et al. (2020) investigated the impact of code review on design degradation (i.e., the progressive worsening of the quality of a system caused by design decisions) (2020). Their findings seem to indicate that presence of design-related discussion in the reviews alone is not enough to reduce design degradation. Similarly to the study of Morales et al. (2015), Uchôa et al. (2020) indicate collaboration among reviewers as a fundamental factor that impacts design quality: e.g., long discussions or a high disagreement among reviewers seem to increase the risk of design degradation.

Focusing on reviewers’ participation, Thongtanunam et al. (2017) investigated the factors that influence reviewers’ participation looking at the characteristics of a review patch Thongtanunam et al. (2017). Among the many factors that impact the likelihood of a patch being discussed, they reported, for instance, the churn of a patch and its description length.

In this study, we focus on both the outcomes of the process and reviewers’ interactions during code review, but we do so by looking at the changes that a review patch undergoes during the code review process. Similarly to the work of Thongtanunam et al. (2017), we analyze factors that might influence reviewers’ participation. However, we do so from a different perspective: We look at factors that lead to a higher presence of review changes triggered by reviewers’ comments in a review change-set.

To the best of our knowledge, only few previous studies (Mäntylä and Lassenius 2009; Beller et al. 2014) assessed the outcomes of code review by looking at review changes. Among them, the most recent is the work of Beller et al. (2014), where the authors analyzed review changes from two open-source projects (ConQAT and GROMACS) using a taxonomy adapted from previous literature (Mäntylä and Lassenius 2009). Furthermore, they investigated the cause of a review change as well as the factors that influence the number of changes in a review change-set.

In our study, we aim to revisit the findings of Beller et al. (2014). To this aim, we consider three new software systems (Android, JGit, and Java-client) and add a further level of granularity to our classification (as defined by Mäntylä and Lassenius 2009), not considered by Beller et al. (2014). We included this finer level of granularity to achieve deeper insight on the outcomes of the code review process. Moreover, we focus on reviews as opposed to *tasks* to obtain a representative image of the review process of the selected projects, regardless of whether the reviews originated from a task or not.

## Methodology

This section describes how we structured our study in research questions, summarizes the steps followed to create the dataset used in our investigation, and presents the method we used to answer each research question.

### Research Questions

The overarching goal of our study is to deepen our empirical understanding of the outcome of Modern Code Review. In recent years, Modern Code Review has become the main standard for reviewing code in industry (Baum et al. 2016; Rigby and Bird 2013). However, previous studies revealed the existence of a significant mismatch between developers and managers’ perception of the outcomes of code review (e.g., catching severe bugs early in the software development cycle) and the real outcomes of this process (e.g., identifying few, low-level bugs) (Bacchelli and Bird 2013; Czerwonka et al. 2015).

To understand the outcomes of code review, we focus our investigation on *review changes*. We structure our investigation around three main research questions. With our first research question, we aim to challenge the findings of previous studies on a new set of open-source projects. In fact, previous work investigated only few projects: two open-source projects (one with only 216 changes) (Beller et al. 2014) or a limited number of industrial and students’ reviews. Given the importance of assessing the outcome of code review, challenging previous findings is critical. Moreover, we aim to deepen our insight in the code review process by including in the classification a finer level of granularity, review changes sub-types, not considered in the study of Beller et al. (2014).

To understand which kind of code changes developers perform during the review process, we start our investigation by constructing a dataset of classified review changes. We classify review changes from three popular open-source projects and answer the following question: 
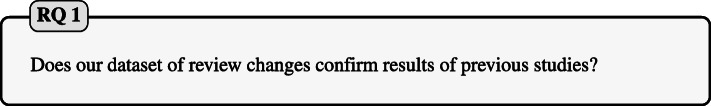


We classify the review changes into the taxonomy refined by Beller et al. (2014) (for category and type level) as well as Mäntylä and Lassenius (2009) (for the sub-type level).

Then, we aim to understand how many of the changes contained in our dataset were caused by a reviewer’s comment on Gerrit. For this reason, we classify the changes is our dataset between those triggered by a comment and those triggered instead by a different cause (e.g., an independent decision from commit’s author, a comment received outside the code review tool environment, or an informal discussion). Similar to RQ1, our aim is to challenge and expand previous findings. We analyze three open-source projects, different from the ones already considered in previous studies. This leads us to the second research question: 
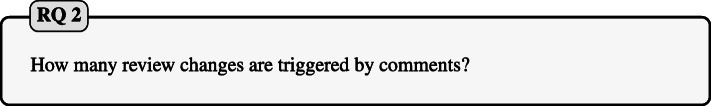


Moreover, we aim to expand our knowledge on code review by looking at the characteristics of the reviews contained in our dataset. For this reason, we investigated which features of a revision might influence the ratio of changes triggered by a reviewer’s action and undocumented changes. While performing this investigation, we notice possible correlations between the number of changes and other characteristics of a review. To test our hypothesis, we finally formulate the following research question: 
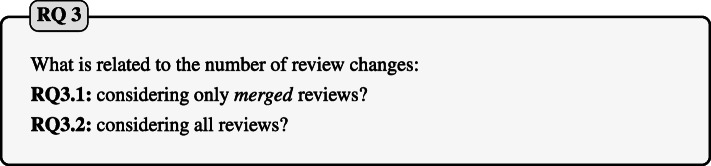


Here our goal is twofold: testing new possible factors correlated to our independent variable and confirm promising results obtained by the previous study conducted by Beller et al. (2014) on metrics such as code churn or the number of files in the initial patch. In **RQ3.1**, we consider only *merged* reviews and we focus on factors used in previous work (Beller et al. 2014) to corroborate previous findings. In **RQ3.2** instead, we expand on previous work by considering new factors (e.g., number of bots), not considered by previous studies, that might have an impact on the number of changes in a review on Gerrit. Moreover, we considered not only *merged* reviews but all kinds of reviews contained in the review history of the projects under analysis.

### Dataset

To create the dataset used to answer our research question, we consider three aspects: subject systems, subject types of files, and subject reviews.

#### Subject Systems

We consider three open-source projects that use Gerrit as code review tool. One is the heavily investigated Android project (which has been proven representative of the code review process in open-source projects and has many active developers and reviewers (Pascarella et al. 2018; Bavota and Russo 2015; McIntosh et al. 2014; 2016)) and the remaining two are part of the CROP dataset (Paixao et al. 2018): JGit and Java-client. We select these projects because they (1): (2) are representatives of the two project communities in the CROP dataset (Eclipse and Couchbase), (3) contain the highest number of reviews and revisions among the Java-based projects in each community.

We focused on Gerrit as it is a popular code review platform used by many major software projects (Pascarella et al. 2018). Other than Android, Gerrit is used, for instance, by OpenStack and QT: Projects that have been previously validated as valuable subjects of studies for investigations of the code review process in open-source projects (Bavota and Russo 2015; McIntosh et al.^2014^, ^2016^). Moreover, most open-source projects using Gerrit offer publicly available code review repositories and an API to allow the extraction of review data. Both these characteristics are fundamental for the creation of our dataset.

#### Subject Types of Files

In the first project (Android), we consider different types of files (e.g., .h, .cc, .java, .go) to obtain a set of changes as diverse as possible. However, despite the good level of agreement reached by the authors, the classification of review changes at sub-type level was prone to errors, requiring an inflated level of understanding of the code. Therefore, to ensure the soundness of the dataset, we focused only on Java-based projects. We aimed to reduce potential bias in the classification caused by the different level of expertise of the authors with different programming languages. In fact, the authors who conducted the classification have a higher level of expertise in Java compared to other programming languages. Therefore, in JGit and Java-client we only analyze Java files.

#### Subject Reviews

Gerrit assigns a unique identifier (ID) to each review. To construct the dataset, we randomly extract an ID among all review IDs for the selected project. Once a review is selected, we check that it is status is *merged*. We focus only on merged reviews to obtain a representative sample of the review process of the project. Then, and we randomly select a patch among all the different patch sets contained in the review. We analyze the difference with the previous patch to extract code changes. We do not consider the first patch in a review since we are not interested in changes introduced *before* the code review process started. We take into account all the files contained in the selected patch set. The dataset of classified review changes is available online.^2^ The information necessary to build the dataset was collected using the Java implementation of the gerrit REST API.^3^ The data collection and subsequent manual labeling process took place between March and August 2019.

### RQ1 (Types of Review Changes) - Research Method

Using the previously described dataset, we classified each review change into a category and type according to the taxonomy introduced by Mäntylä and Lassenius (2009) and later refined by Beller et al. (2014), and into a subtype according to the taxonomy introduced by Mäntylä and Lassenius (2009), as these were not considered in Beller et al. (2014)’s version of the taxonomy. Compared to the study of Beller et al. (2014), we assign each change to a *subtype* to gain deeper insight into the code review process. Such granularity level was already used in the study of Mäntylä and Lassenius (2009) but applied only to a small set of industrial and students’ reviews. In this study, we instead apply this finer-grained classification to a large amount of reviews from popular open-source projects.

The first author of this manuscript conducted this manual classification of the review changes. To support the classification task, we built a tool that displays a selected review patch-set. As manually classifying review changes is a repetitive and time-consuming task, a tool can support this process and reduce possible errors. We describe here the tool to illustrate the steps taken in the dataset creation and to increase the confidence in the methodology and the results of this investigation. The tool first selects a random review ID, then a random patch among the ones belonging to the review (following the methodology reported in Section 3.2). Information on the review (e.g., review ID, patchset IDs, and code) is extracted from the Gerrit online repository of the selected project using a Java implementation of the Gerrit REST API.^3^ Through this API it is also possible to retrieve the diff between the old and the new patches in a review.

We manually assigned a category, type, and subtype to each change in the selected patchset. In a second step, we linked each change in the dataset to its corresponding code snippet as displayed in Gerrit (this process was done automatically and manually verified by the first author). The code of the classification tool is available in our replication package.^2^

**Fig. 16:**
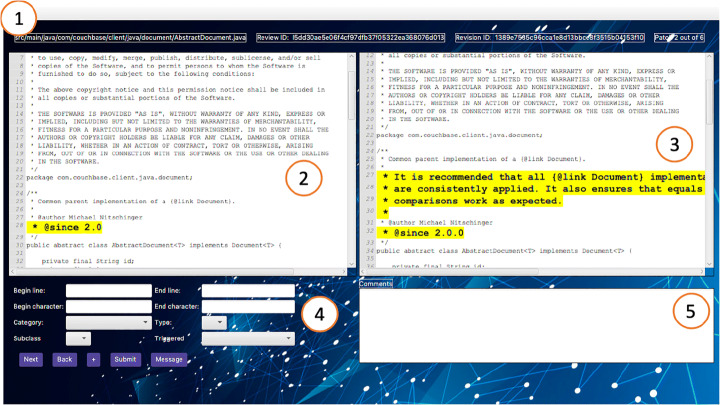
Review changes classification tool

Figure 16 shows the main page of the tool used to classify review changes. Part contains general information: the name of the file under analysis, the review and patch set ID, and the patch number (e.g., patch 2 out of 6). To allow users to classify changes, the tool shows the code contained in the selected patch set (part in Fig. 16) and the one contained in the previous patch set (part in Fig. 16), highlighting the difference between the two versions of the file. A panel (Part ) is used to classify a change, while a box (part ) displays any reviewer comment (if any) associated with the file. Moreover, the user can also visualize the commit message associated with the review. This message will be displayed in a pop-up window.

**Fig. 17:**
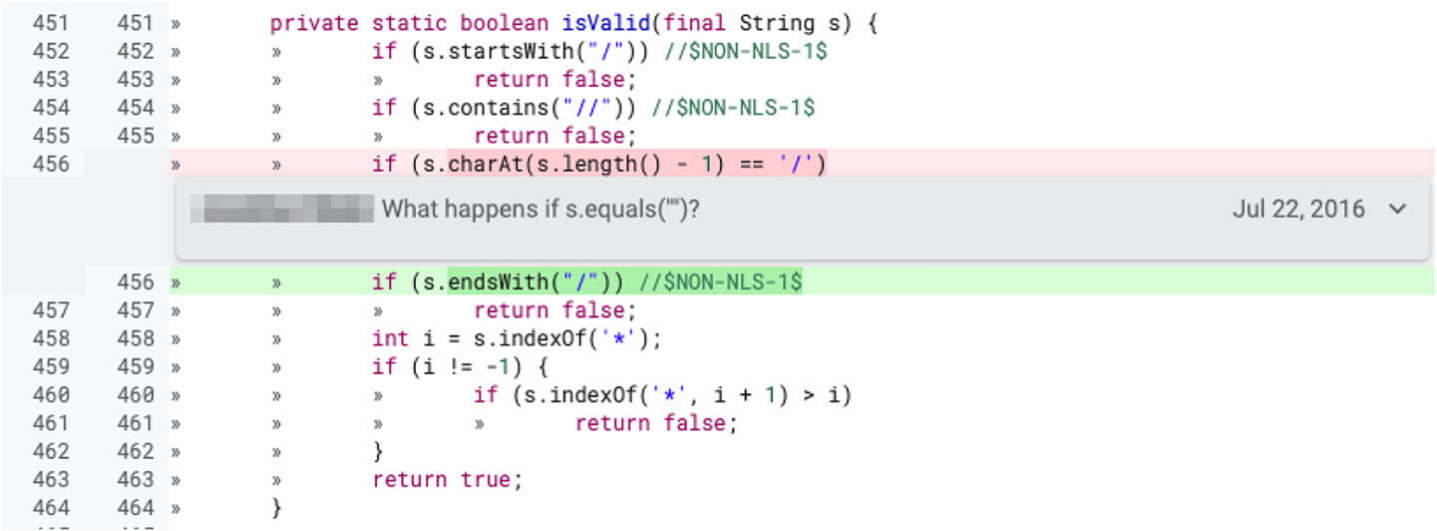
Example of a functional change that also involved a maintainability (evolutionary) change. While changing the logic of an *if* statement, the developer also added a comment to complete the change. The real-world code review in which this change took place is available online (https://git.eclipse.org/r/c/jgit/jgit/+/77733/2..3/org.eclipse.jgit/src/org/eclipse/jgit/transport/RefSpec.java, July 2016.)

In accordance with the strategy adopted in previous work (Mäntylä and Lassenius 2009; Beller et al. 2014), in case a change could be classified as functional or evolutionary, we gave priority to the former. This choice was adopted to take into account the more severe consequences that functional changes entail. Moreover, functional changes often involve maintainability issues. For instance, Fig. 17 shows a *Logic* functional change that also involved the addition of a comment.

Even though we follow a methodology similar to the one of Beller et al. (2014), our study focuses on reviews as opposed to *tasks* (i.e., project’s issue). In fact, Beller et al. (2014) first randomly selected a subset of tasks and then linked them to the corresponding reviews; instead, in our approach, we directly select a subset of reviews from the Gerrit repository of each project. Our goal is to obtain an image as representative as possible of the kinds of changes that happen during code review. Therefore, we are not focused on knowing whether these reviews were originated from a task (or similar issues instances) or not.

#### Logically Related Code Changes

**Fig. 18:**
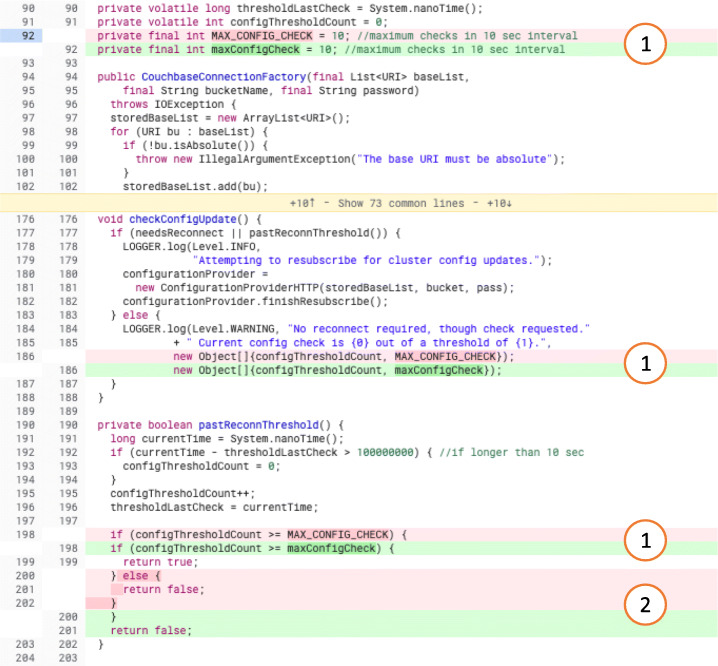
Example of logically related code changes. Line 92 shows the renaming of a variable from *MAX_CONGFIG_CHECK* to *maxConfigCheck*. The same renaming took place at lines 186 and 198. These three changes are logically related to each other, therefore we consider them as a single change. The change at lines 200-202 is instead unrelated to the previous ones and, therefore, we treat it as a separate change. The real-world code review in which this change took place is available online (http://review.couchbase.org/c/couchbase-java-client/+/14574/1..2/src/main/java/com/couchbase/client/CouchbaseConnectionFactory.java, April 2012)

To avoid issues with the presence of the same kind of modification in multiple changes, we decided to consider these cases as a unique change. Therefore, we considered as a single change multiple code chunks logically related to each other. For example, considering the case of a variable renaming when there are multiple occurrences of the same variable spread across the file, not counting all these occurrences as a single change might lead to overcounting certain kinds of changes compared to others. Figure 18 shows an example of such an occurrence. In the file under analysis, three changes (marked as ) involve the renaming of the same variable *MAX_CONFIG_CHECK*. Therefore, we treat them as a single change (assigning to all three changes the same ID). The change marked as  is instead unrelated to the renaming of *MAX_CONFIG_CHECK*. For this reason, we consider it as an independent change (giving it a unique ID). This feature is supported by the devised classification tool.

#### Changes to the Taxonomies

Although the selected taxonomies Mäntylä and Lassenius (2009) and Beller et al. (2014) cover almost all the cases we encountered, we slightly modified the taxonomies to solve situations of uncertainty in our classification. In particular, we extended the *Move functionality* sub-type to include cases where a functionality was moved across the same class (e.g., statements moved from a method to another) and the *Data & Resource Manipulation* to include functional issues concerning wrong variables assignments. The changes that we could not classify with sufficient confidence were marked as *Unknown*. Table 1 reports the number of classified and *Unknown* changes included in our dataset for each of the three projects considered.

**Table 1:** Number of changes in the dataset for each project

Project	Classified changes	Unknown changes	Total changes
Android	505	80	585
Jgit	502	80	582
Java-client	503	56	559
Total	1,510	216	1,726

#### Classification Validity

To verify the correctness of the classification, the second author of this manuscript independently classified a subset of the changes, then we compared the results. In detail, the second author classified a statistical significant subset of 306 changes, computed with a confidence level of 95% and a margin of error of 5%. In this subset, the inter-rater percent agreement was 90.52% at category level, 73.87% at type level, and 64.7% at subtype level. Moreover, we computed the inter-rater agreement using Krippendorff’s Alpha (Krippendorff 2011)^4^ obtaining the following results: 0.457 at category level, 0.657 at type level, and 0.6 at subtype level. The alpha value at category-level reports only a moderate agreement. However, this reflects the intrinsic unbalance in our dataset, where evolvability changes represent the vast majority of code review changes, as reported by previous studies (Beller et al. 2014; Mäntylä and Lassenius 2009). The changes where the two authors disagreed were the object of further discussion. Once an agreement on their classification was found, these modifications were included in the dataset. Changes that the first author could not classify with sufficient confidence were classified as ‘unknown’ and disregarded in this validation process as well as in the subsequent analysis.

### RQ2 (Triggers for Review Changes)—Research Method

For each review change in our dataset, we check whether the change was introduced as a consequence of a comment from a reviewer. We consider in-line comments added directly to the code during the code review process (e.g., Fig. 12) and general comments written directly in the review task. If a change is caused by a reviewer’s observation, we report it in our dataset as ‘triggered by a comment’. Whenever a review change cannot be linked to a previous reviewer’s comment, we classify it as ‘undocumented’. In this phase, we label not only the 1,510 changes to which we assigned a category and type in our RQ1 but also the 216 changes previously marked as “Unknown”. This led to the creation of a new dataset containing 1,726 changes (as reported in Table 1). In Android, we considered all the files contained in the selected revisions, regardless of their type.

#### Labeling Validity

We computed a statistically significant subset of the changes in our dataset with a confidence level of 95% and an error margin of 5%. This led to the creation of a subset of 315 changes that were independently labeled by the second author. Then, we computed the inter-rater agreement on this subset of 315 changes among the authors involved in the labeling. This process led to an inter-rater percent agreement of 91.11%. Moreover, we computed Krippendorff’s Alpha obtaining a value of 0.8. Krippendorff’s Alpha can assume values from 0 to 1, where 1 is the perfect agreement. Therefore, a value of 0.8 indicates a strong agreement between the raters. Any conflict arising was solved through a discussion among the authors involved in this process. The modifications to the labeling of the changes, obtained as result of the discussion, were included in the dataset.

#### Triggered/Undocumented Changes Ratio

Table 2 shows the model (dependent and independent variables) we consider in the study of the factors related to the number of review changes. Code churn, author, and number of files have been chosen as variables to challenge previous results in the field. The other metrics have been selected to test new relationships. In particular, we hypothesize that the size of the patch under review might influence the number of changes done during the review process. We included lines added and lines deleted, together with churn to allow for a finer granularity of our analysis. For instance, reviewers might disregard lines of code deleted when performing a review, preferring to focus instead on the new content added in the patch.

**Table 2:** Model considered in the investigation of RQ2

Dependent	Description	Rationale
variable		
Ratio	Number of triggered changes	The number of undocumented
	divided by the total number of	(documented) changes in a
	changes (triggered and	revision might be influenced by
	undocumented) in a revision.	some specific characteristics of
		the revision itself.
Independent	Description	Rationale
variable		
Code churn	Total number of added and	Larger changes might have more
	deleted lines of code.	reviewers’ comments leading to
		a change.
Author	The author of the code under	Some authors might adopt more
	review.	changes without being prompted
		by a reviewer.
Number of files	Number of files in the initial	A modification that touches a
	review patch.	large number of files might be
		more prone to defects, leading to
		an increase number of change-
		inducing comments left by reviewers.
Lines added	Lines of code added in the	A higher number of lines of code
	initial patch.	added could increase the amount
		of reviewers’ comments asking for
		fixes.
Lines deleted	Lines of code deleted in the	Deleting code lines might
	initial patch	introduce defects in the code.
		Therefore, a change-set with many
		deleted lines might lead to more
		reviewers’ remarks pointing to
		issues in the code.
Size	Total number of code lines in	Introducing modifications large
	the files in the initial patch.	files might be challenging,
		leading to a higher number of issues
		marked by reviewers.

#### Subject Revisions

We consider the three projects contained in our dataset (i.e., Android, JGit, and Java-client). We developed a script to extract the data for our statistical model from the Gerrit repository of each project by leveraging the Java client of the Gerrit REST API.^5^ We extracted the features for 544 revisions (201 for Android, 210 for JGit, and 133 for Java-client) from the 552 revisions contained in our dataset. For the remaining eight revisions, we could not extract the data from Gerrit because of issues in the Gerrit repositories of the projects. However, to strengthen the soundness of our findings, when classifying each review change, we did not rely exclusively on the information retrieved using the REST API. Before classifying each change, the authors checked the context of each review change (e.g., global discussions among reviewers or title of the Pull Requests under review) on the Gerrit repository of the project and used this information to label the changes. Furthermore, the authors inspected previous patches for comments that could have triggered a change only few patches later (and not in the immediately subsequent patch). Only changes that could not be clearly traced back to a reviewer’s comment were classified as ‘undocumented’.

#### Statistical Modeling

To analyze the relationship between the explanatory variables and the triggered/undocumented changes ratio, we built a logistic regression model. While building the model, we checked the collinearity among explanatory variables computing the Variance Inflation Factor (VIF) (Fahrmeir et al. 2013) and removed the one with the highest VIF among all of those with VIF above five: Values of VIF greater than four are signs of severe multicollinearity (Craney and Surles 2002; O’Brien 2007). We applied this process iteratively on the remaining variables until no variables with VIF above the threshold are left. Since the variable *Author* is a categorical variable having more than one degree of freedom (it can independently assume the value of one of the reviewers that took part in the considered reviews), we used the computation of the Generalized Variance Inflation Factor (GVIF) (Fox and Monette 1992): When one or more variables included in the model have a degree of freedom higher than one, we can not employ the standard VIF but we must resort to its generalized version (GVIF). To employ the same exclusion thresholds generally used for the standard VIF (in our case, five), we must compare the (*GV* *IF*^(1/(2∗*Df*))^)^2^ (where *D*_*f*_ is the degree of freedom of the considered variable) value of the variables included in our analysis (Buteikis 2020). Variables with (*GV* *IF*^(1/(2∗*Df*))^)^2^ higher than five must be removed from the model. The script used to compute the (*GV* *IF*^(1/(2∗*Df*))^)^2^ and to conduct our analysis is available in our replication package.^6^ Overall, we created a total of four logistic regression models: one for each project (Android, JGit, and Java-client) and one including revisions from all three projects.

We built a model for each of the three considered projects to investigate the impact of each feature included in our model. Our aim is to understand which features are important in all projects and, on the contrary, which features play a key role only in a specific scenario. For instance, the importance of the features in predicting our dependent variable might be influenced by the specific review practices of a project. Moreover, we built a general model with all three projects to (1) mitigate potential bias introduced by the specific characteristics of a project and (2) allow us to understand if the conclusions drawn for a single project still hold true in a more general scenario.

### RQ3.1 (Relations to the Number of Review Changes)—Research Method

In RQ3, our aim is to verify if the number of changes happening during a review might be influenced by specific characteristics of the review itself (e.g., size of the files to be reviewed). For this reason, we consider as dependent variable the *number of review changes*: The amount of changes to which the code under review undergoes before the end of the review process. While in RQ2 we focused only on the changes contained in our dataset, here we expand the number of considered review changes because our analysis does not rely anymore on manually created labels. Table 3 shows the independent and dependent variables considered in our investigation. We employed variables already applied to RQ2. However, differently from what was done in RQ2, we include in our model also the number of reviewers who took part in the review (similarly to what done by Beller et al. (2014)) and the cyclomatic complexity of the changes. These variables were not possible to extract in the context of our previous research question. The Gerrit rest API does not allow to extract the number of reviewers present in a specific point of the review, making it impossible to adopt this metric at revision-level. Concerning cyclomatic complexity, the Android project is written in many different programming languages, which would have made the complexity calculation extremely challenging. At the same time, we could not limit our analysis to only some specific programming languages without significantly reducing the size of our sample.

**Table 3:** Model considered in the investigation of RQ3

Dependent	Description	Rationale
variable		
Number of review changes	Number of changes to which	The number of changes
	the code under review	happening during review
	undergoes before the end of	might be influenced by specific
	the review process.	characteristics of the review (e.g., size of
		the files to be reviewed).
Independent	Description	Rationale
variable		
Author	The author of the code under	Some authors might need more
	review.	review changes (Beller et al. 2014)
Complexity	The McCabe cyclomatic	A review containing changes
	complexity	with high complexity might lead
		to more potential mistakes that
		need to be fixed during the
		review.
Number of reviewers	Number of reviewers	Having a higher number of
	allocated to the review.	reviewers involved in the review
		might lead to a higher number
		of changes.
Control	Description	Rationale
variable		
Code churn	Total number of added and	Larger changes tend to require
	deleted lines of code.	more review changes
		(Beller et al. 2014).
Number of files	Number of files in the	A modification that touches a
	initial review patch.	large number of files might be
		more prone to defects, leading
		to subsequent changes
		(Beller et al. 2014).
Lines added	Lines of code added in	The higher the number of lines of
	the initial patch.	code added, the higher the
		possibility that the code will need
		further fixing.
Lines deleted	Lines of code deleted in	A vast number of deleted lines
	the initial patch	has a higher chance to contain
		lines removed by mistake.
Size	Total number of code lines	Introducing modifications in
	in the files in the initial	files having a high number of
	patch.	lines of code might be
		challenging, therefore leading to
		possible issues to be solved at
		review-time.

Moreover, the variables lines added, lines deleted, and churn act as control factors on the goodness of our model. We expect these variables to be correlated with the number of review changes as, for instance, a review change-set with a high number of lines changed is more likely to have received more comments from reviewers.

Furthermore, while in RQ2 our focus was at *revision* level (Section 2.1), in RQ3 we work at the level of *reviews*, which might be composed of multiple revisions.

#### Subject Reviews

We consider the three projects used in our previous analysis (i.e., Android, JGit, and Java-client). We used a script similar to the one employed in RQ2 to extract the data for our statistical model. Since Java is the main language of two of the three projects included in our dataset (JGit and Java-client), we restricted our analysis to reviews containing only Java files. Restricting our analysis to only one programming language allowed us to compute the cyclomatic complexity of the changes and include it as metric in our analysis. Furthermore, we filtered out reviews with only one patch and excluded reviews that were not merged into the code-base. We focused only on merged reviews to avoid introducing bias in our analysis: e.g., *open* reviews might still undergo further review iterations, potentially increasing the number of review changes.

We counted as *review change* a group of contiguous modified lines of code between two different review patches (as shown in Fig. 1). This offers a finer granularity level (compared to instance to the number of patches), which, in return, allows us to collect deeper insights on the code review process. Other variables (e.g., the number of review patches) might not have been a good indicator of the real number of changes a pull request underwent (e.g., a single patch can contain an arbitrary number of changes.)

We collected all the reviews according to the aforementioned criteria, using their Gerrit review ID (i.e., the unique code associated with each review in Gerrit). For each project, we used the following ID intervals: [0, 100’000] Java-client, [1, 155’000] JGit, and [1, 800’000] Android. We collected 646 samples from Java-client, 2,580 from JGit, and 16,430 from Android. We selected large ID intervals to include in our dataset changes sampled across many years of the project review history. Android data showed invalid entries caused by issues in mining the Gerrit repository of the project. We removed these 479 reviews from our dataset. The final Android dataset contained 15,951 entries and the whole dataset 19,177 reviews. We considered only a randomly selected sample of 5,000 instances in Android to simplify the comparison of the results across the three projects and avoid introducing bias in the model that includes all three projects because of the significantly higher number of reviews in Android. Moreover, we included in this investigation also reviews that did not lead to any change. The Java-client sample contains 383 instances of reviews not leading to any changes, the JGit sample 927, and the Android sample (limited to the first 5,000 instances) 1,264.

#### Statistical Modeling

To analyze the relationship between the explanatory variables and the number of changes, we use a Generalized Linear Model (GLM). This model explains retrospectively, using a set of explanatory independent variables, the characteristics of a dependent variable (in our case, the number of review changes). Moreover, a GLM can handle both cardinal and discrete variables (Beller et al. 2014), making it the ideal choice based on the independent variables we selected.

As first step in our analysis, we evaluated which distribution best fits our dependent variable. We found that our data could be well represented with a Poisson distribution (this is true for all three projects and the whole dataset). To apply GLM, we modeled the number of changes as a binomial distribution, following the guidelines given in previous studies (Beller et al. 2014; Krebs 1999). To prepare the model, similar to what was done in RQ2, we checked the collinearity among explanatory variables computing the General Variance Inflation Factor (GVIF) (Fahrmeir et al. 2013) (since the variable Author is a categorical variable having more than one degree of freedom). We removed the variable with highest (*GV* *IF*^(1/(2∗*Df*))^)^2^ among all of those with (*GV* *IF*^(1/(2∗*Df*))^)^2^ above five. We repeated this process until no variables with (*GV* *IF*^(1/(2∗*Df*))^)^2^ above five were left. Furthermore, we removed aliased variables from our analysis. Overall, we created a total of four GLM models: one per project and one for all the reviews across all projects.

### RQ3.2 (Beyond Only *merged* Reviews)—Research Method

Our previous investigation confirmed and extended the results of Beller et al. (2014) on a different set of projects using a similar set of metrics as the ones used in the original work. Given the good results achieved by this initial investigation, we further extend these findings. To do so, we consider JGit and two new java-based projects (also represented in the CROP dataset (Paixao et al. 2018)). We add two new projects (egit and eclipse platform ui) to further generalize our findings on a larger set of projects. We consider all reviews associated to the specific project and containing only java files having IDs in the range [1 : 200’000].

In this investigation, we do not focus only on *merged* reviews but on all kinds of reviews contained in the projects (e.g., *abandoned* reviews) to evaluate if our findings can be extended beyond only merged reviews. Moreover, we extend the set of the considered independent variables with new variables that might be related to the number of changes in a review. First, we include in the model the status of the review (e.g.,. merged). Our hypothesis is that merged reviews might have undergone a higher number of changes before getting approved, while other kind of reviews might have been immediately discarded.

Then, we include as variables (1) the topic and (2) branch of the review change-set as, for instance, a specific topic might require more attention from reviewers (which might lead to a higher number of changes). We also include the experience of the author and the average experience of the reviewers based on the number of reviews they took part in. In fact, more experienced reviewers might suggest a higher number of changes while, on the contrary, the code authored by more experienced developers might require significant less modifications at review time. Moreover, inspired by the work of Thongtanunam et al. (2017), we include in our model also (1) the number of directories in the review change-set, (2) the length of the review description, and (3) the length of the subject of the review. For instance, previous findings revealed how change-sets with a well explained change log message draw more attention of reviewers (Rigby and Storey 2011).

Finally, we focus on the number of bots added to the review. Previous findings highlighted how the presence of bots can significantly change the dynamics of developers’ collaboration (Wessel et al. 2020). For this reason, we decided to include this variable in our model. Our hypothesis is that bots are natural triggers of changes as they suggest to the author issues with the submitted review patch-set.

Following the methodology of RQ3.1, to conduct our analysis we build four Generalized Linear Models (GLMs), one for each project and a total one that includes all three projects.

## Results

In this section, we present the results by research question.

### RQ1—The Types of Review Changes

The overall ratio between ‘Evolvability’ and ‘Functional’ changes is approximately 90:10 with a difference of less than two percentage points. The results are stable across the three projects. In particular, Android presents 88.9% evolvability changes, as opposed to 11.1% functional changes; JGit has 90.8% evolvability changes and only 9.2% functional changes; finally, in Java-client 89.7% of the changes are evolvability changes, while 10.3% belong to the functional category.

**Fig. 19:**
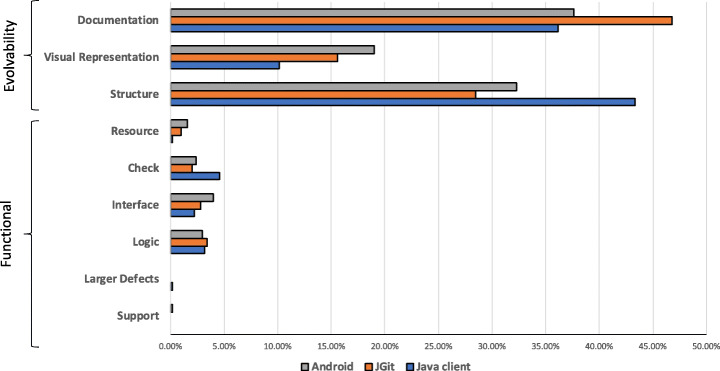
Distribution of review change types by project

Figure 19 shows the percentage of changes, divided by type, in each of the projects under analysis. ‘Textual’ and ‘Supported by language’ changes have been grouped together in an intermediate category ‘Documentation’. The same applies to the ‘Organization’ and ‘Solution approach’ changes, which have been clustered in the ‘Structure’ category of review changes.

‘Documentation’ changes are the majority in both JGit and Android, but they are exceeded by ‘Structure’ changes in Java-client (44% structure vs. 35% documentation changes). Regarding functional changes, in Java-client there is a clear majority of ‘Check’ changes, while in the other two projects the difference between the percentage of ‘Check’, ‘Interface’, and ‘Logic’ review changes is limited.

Table 4 shows the distribution of ‘Evolvability’ changes in the three systems under analysis. ‘Textual’ review changes are the majority of the ‘Evolvability’ changes in Android and JGit. However, in Java-client ‘Solution approach’ review changes represent the majority of changes in our dataset. Table 5 shows the distribution of ‘Functional’ changes in each project. ‘Interface’ changes represent the majority of functional changes in Android. However, they are less prominent in Java-client, where the majority of functional changes belongs to the ‘Check’ type, and in JGit, where most prominent changes type is ‘Logic’. In our investigation, we identified only a few occurrences of ‘Support’ and ‘Larger defects’ changes. This might depend on the nature of these changes that involve major issues, e.g., a functionality left partially unimplemented, (Larger defects) or problems with support systems or libraries (Support). We argue that, by definition, the number of these changes is already limited.

**Table 4:** Distribution of ‘Evolvability’ review changes by type and project

Review change type	Android	JGit	Java-client
Textual	30.7%	43.0%	28.8%
Supported by language	6.9%	3.8%	7.3%
Visual Representation	19.0%	15.5%	10.1%
Organization	9.1%	8.0%	8.0%
Solution approach	23.1%	20.5%	35.4%

**Table 5:** Distribution of ‘Functional’ review changes by type and project

Review change type	Android	JGit	Java-client
Check	2.4%	2.0%	4.6%
Interface	4.0%	2.8%	2.2%
Logic	3.0%	3.4%	3.2%
Resource	1.6%	1.0%	0.2%
Support	0.2%	0%	0%
Larger defects	0%	0%	0.2%

Each type can be further divided into sub-types. To gain deeper insight into the code review process, we extended our analysis to this level. As expected from the analysis at type-level, the majority of the changes belong to sub-types of the ‘Documentation’ and ‘Structure’ classes. Android and JGit have a prevalence of review changes impacting comments, naming of software elements, and implementation of new functionalities (*new functionality changes*). In Java-client the naming changes do not have the same relevance as in the other projects; instead, ‘change function’ changes represent the third most frequent subtype of changes.

Table 6 shows the distribution of the subtypes of ‘Evolvability’ changes in our dataset, divided by type. A description of each subtype is available in the online material.^7^ Among the ‘Textual’ changes, the majority is represented by comments in the code. This tendency holds for all three projects in our dataset. ‘Naming’ changes, which involve problem with software elements’ names, have a different impact depending on the project under analysis: They account for the 37% of textual changes in Android, while only for the 18% and 15% in JGit and Java-client, respectively. Finally, almost no instances of ‘Debug info’ review changes were found in our analysis.

**Table 6:** Distribution of evolvability changes based on their subtype and grouped by type. A description of each subtype is available in the online material (10.5281/zenodo.6811165)

Review change type	Sub-type	Distribution	
Textual	Naming	119	23.1%
	Comments	355	68.8%
	Debug info	2	0.4%
	Others	40	7.7%
	*Total*	*516*	*100.0%*
Supported by language	Element Type	38	41.7%
	Immutable	27	29.7%
	Visibility	20	22.0%
	Void parameter	0	0.0%
	Element reference	6	6.6%
	*Total*	*91*	*100.0%*
Visual representation	Bracket usage	22	9.8%
	Indentation	38	16.9%
	Blank line usage	90	40.0%
	Long line	33	14.7%
	Space usage	24	10.6%
	Grouping	18	8.0%
	*Total*	*225*	*100.0%*
Organization	Move functionality	35	27.8%
	Long sub-routine	0	0.0%
	Dead code	40	31.7%
	Duplication	7	5.6%
	Complex code	2	1.6%
	Statement issues	22	17.5%
	Consistency	5	3.9%
	Others	15	11.9%
	*Total*	*126*	*100.0%*
Solution approach	Semantic duplication	7	1.8%
	Semantic dead code	35	8.8%
	Change function	80	20.1%
	Use standard method	30	7.5%
	New functionality	208	52.0%
	Others	29	7.3%
	Minor	10	2.5%
	*Total*	*398*	*100.0%*

The rows in italics represent the total number of changes in each category (e.g., Textual)

The majority of ‘Supported by language’ changes are ‘Element type’ changes: changes in which the element was of a wrong type (not causing any runtime error). This remains true even when considering only the changes related to Android and Java-client. However, in JGit the most represented subtype (63%) is the ‘Immutable’ subtype. ‘Element type’ changes account for only 10%. Furthermore, we detected a small presence of visibility changes in all three projects (17.1%, 26.3%, and 24.3%, respectively).

The use of blank lines (‘Blank line usage’ changes) and the indentation issues are the most important reason behind changes in the visual representation type. In particular, ‘Blank lines’ review changes are the majority of visual representation changes in all the three projects under analysis. Concerning organization changes, the two most prominent subtypes are removals of portions of dead code (‘Dead code’ changes) and reorganization of functionalities across the code-base (‘move functionality’ changes). These two subtypes represent the majority of changes in Android and Java-client, but in JGit they are outnumbered by statement issues changes.

Finally, the vast majority of ‘Solution approach’ review changes involve the implementation of a new functionality to make the code more evolvable (i.e., ‘New functionality’ changes). This applies across projects. Changes originated by the need to change a function in favor of a better alternative (i.e., ‘Change function’ changes) define a large part of the ‘Solution approach’ review changes in our dataset across all projects.

Table 7 shows the distribution of all functional subtypes across the three considered projects. The two most common sub-types are ‘Check variable’ review changes and ‘Parameter’ review changes. The former are modifications that introduce a check on a variable, while the latter are changes that fix missing or incorrect parameters in a function call. These two subtypes of changes constitute the majority of ‘Functional’ review changes across all projects.

**Table 7:** Distribution of functional changes based on their subtype and grouped by type. Support changes do not possess subtypes. A description of each subtype is available in the online material (10.5281/zenodo.6811165)

Review change type	Sub-type	Distribution	
Check	Check variable	42	93.6%
	Check function	3	6.7%
	*Total*	*45*	*100.0%*
Interface	Parameter	40	88.9%
	Function call	5	11.1%
	*Total*	*45*	*100.0%*
Logic	Compare	28	58.3%
	Compute	2	4.2%
	Wrong location	5	10.4%
	Algorithm & Performance	6	12.5%
	Other	7	14.6%
	*Total*	*48*	*100.0%*
Resource	Data & resource manipulation	12	85.7%
	Variable initialization	2	14.3%
	*Total*	*14*	*100.0%*
Support		1	100%
	*Total*	*1*	*100.0%*
Larger defects	Completeness	1	100%
	*Total*	*1*	*100.0%*

The rows in italics represent the total number of changes in each category (e.g., Textual)

### RQ2—What Triggers Review Changes

#### Distribution of Review Changes

**Fig. 20:**
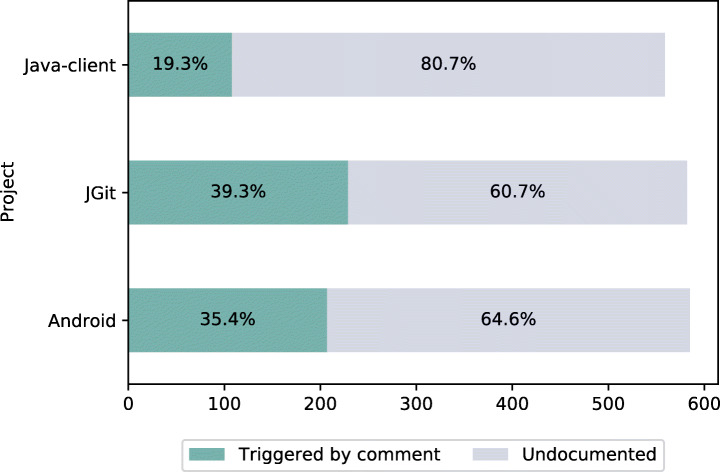
Distribution of review changes: triggered by a comment vs. undocumented

Figure 20 shows the distribution of review changes as triggered either by a reviewer’s comment or not (i.e., undocumented). In our dataset, we find that more than 60% of the review changes are of the undocumented type. This result is in contrast with the findings by Beller et al. (2014), who, instead, reported that a vast majority of the changes were triggered by an explicit request of a reviewer. This difference might have been originated from the different sets of projects considered: conQAT and GROMACS against Android, JGit, and Java-client. For instance, differently from the other projects considered, conQAT adopts a review-after-commit workflow. This might have limited the amount of “spontaneous” changes made by the author of the review change-set. Furthermore, conQAT did not rely on Gerrit, but reviewers used Eclipse instead to assess the code under review. This might have also been a factor that prevented the author of a patch-set to make further changes without the explicit input of a reviewer. Concerning GROMACS, the difference in the number of “triggered by comment” changes between this project and the three systems we considered in our investigation might reside in its community and review policies.

Moreover, our investigation focused on the review practices of developers in Gerrit. Therefore, we marked as *undocumented* those changes for which we could not identify a cause in the developers’ discussions or comments on this code review platform. Our investigation revealed how the majority of review changes are not discussed on Gerrit. This specific focus of our study might have also been another reason of discrepancy with the findings of Beller et al. (2014). We considered only the information exchanged over Gerrit to increase our understanding of how this platform is used by developers and evaluate its feasibility as potential source of information for the development of code review analytics tools.

#### Change Cause by Category & Type

We investigated the correlation between the cause of a change (comment or undocumented) and its category and type. We performed *Pearson Chi-square* tests on our dataset of changes, not considering the ones whose category has been marked as unknown. We tested our hypothesis of correlation on each project separately and on the dataset composed by all of them.

Table 8 shows the results of our correlation tests in terms of Chi-square value (*χ*) and *p*-value between the category of a change (evolvability or functional) and its cause and the type of a change and its cause. A *not valid* entry means that more than 20% of cells had an expected count of less than 5.

**Table 8:** Correlation analysis results of *Pearson’s Chi Square* test on the cause of a change and its category and type, respectively

Project	Cause vs. Category	Cause vs. Type
Android	*χ*(1) = 0.602 *p* = 0.438	Not valid
JGit	*χ*(1) = 3.15*e*^− 5^ *p* = 0.995	*χ*(8) = 7.138 *p* = 0.522
Java-client	*χ*(1) = 1.618 *p* = 0.203	Not valid
All	*χ*(1) = 0.010 *p* = 0.919	*χ*(10) = 27.192 *p* = 0.002

In both cases, the *p*-value obtained led us to conclude that there is no statistically significant correlation between these factors. The only exception was obtained using the all three projects: here, we measured a correlation between the type of a change and its cause (*p* − *value* < 0.01). In this case, we checked the strength of the correlation by analyzing its effect size using *Cramér’s V* measure. We obtained an effect size of 0.134, leading us to conclude that there is only a weak association among the type of a change and its cause.

Our findings on the correlation between the cause of a change and its category were further corroborated by performing a *Fisher’s Exact test*. This test gave us the following results in terms of *p*-value for all four cases: 0.388 (Android), 0.877 (JGit), 0.153 (Java-client), and 0.858 (All).

#### Analysis of Ratio of Triggered/Undocumented Review Changes

We investigated the correlation between the ratio between triggered and undocumented changes and the characteristics of a revision (independent variables in Table 2). The ratio can assume a value of 0 when no changes triggered by comments are contained in the revision. Our dataset includes 293 such cases. To this aim, we created four logistic regression models, one for each project and a global one considering all three projects. In all models, we performed a multi-collinearity analysis to remove highly correlated variables. To evaluate the variables, we considered (*GV* *IF*^(1/(2∗*Df*))^)^2^, the square value of GVIF, as doing so allows us employ the selection threshold normally used for the standard VIF (e.g., five) (Buteikis 2020). In all models we removed the variable code churn as it was too correlated with the variable lines added. Then, we built our models as follows:

**JGit.**: We checked the multi-collinearity of the remaining variables, revealing that all of them have (*GV* *IF*^(1/(2∗*Df*))^)^2^ lower than 5. After this step, we included the following variables in the model: (1) Author, (2) number of files, (3) lines added, (4) lines deleted, and (5) size. All instances of the variable ‘Author’ had a p-value between 0.9950 and 1. This led us to the hypothesis that the variable ‘Author’ does not have a significant impact on the dependent variable ratio. To verify this hypothesis, we performed a *Chi Square* (Greenwood and Nikulin 1996) test comparing two models with and without the variable ‘Author’. The test achieved *Pr*(*χ*) = 0.1773, which is larger than the significance level of 0.05, therefore confirming that ‘Author’ is indeed a statistically insignificant predictor of our dependent variable. Therefore, we removed it from our model. The model achieved an accuracy of 0.56.
**Java-Client.**: After removing churn from the model, we noticed how lines added is highly correlated with number of files. Therefore, we removed lines added from our model. Before constructing our model, we checked that all remaining variables had (*GV* *IF*^(1/(2∗*Df*))^)^2^ values lower than 5. At this stage, we included the following variables in the model: (1) Author, (2) number of files, (3) lines deleted, and (4) size. The variable ‘Size’ had a significance level of 0.976 in our model. For this reason, similarly to what was done with the variable ‘Author’ in JGit, we tested if ‘Size’ is a statistically significant predictor of the dependent variable. We performed a *Chi-square* test between the two models with and without the variable ‘Size’. We obtained a *Pr*(*χ*) = 0.9756, which confirmed that ‘Size’ is a statistically insignificant predictor and can be removed from our model. Our model achieved an accuracy of 0.74.
**Android.**: In the next step of our model preparation, the variable lines added obtained a (*GV* *IF*^(1/(2∗*Df*))^)^2^ higher than five. For this reason, we removed it from the model. A new analysis revealed no other variable with (*GV* *IF*^(1/(2∗*Df*))^)^2^ above five. So, we included the following variables in the model: (1) Author, (2) number of files, (3) lines deleted, and (4) size. However, we noticed that the variable Author presented only instances with p-value equal to or above 0.999. To verify if ‘Author’ is indeed a statistically insignificant predictor of the dependent variable ratio, we performed a *Chi Square* test comparing two models with and without this variable. The test obtained a *Pr*(*χ*) = 0.05998, allowing us to safely remove the variable Author from the model. Our model achieved an accuracy of 0.64.
**Total.**: After removing the variable churn, all remaining variables did not present a significant correlation with each other. Therefore, we included the following variables in the model: (1) Author, (2) number of files, (3) lines added, (4) lines deleted, and (5) size. The final model is reported in Table 9. It was not possible to compute the GVIF of the variables in the model because the variable Author has too many instances compared to the size of the dataset. We noticed that the variable ‘Author’ presented only occurrences with p-value equal to or above 0.998. For this reason, we performed a *Chi Square* to verify if ‘Author’ is a statistically significant predictor of the dependent variable Ratio. We compared two models, with and without the variable ‘Author’. The test resulted in a *Pr*(*χ*) = 0.01598, which is lower than the significance level of 0.05. Therefore, we could not remove ‘Author’ from the model. Our model achieved an accuracy of 0.70.

**Table 9:** Logistic regression models for the three projects separately and the complete dataset. Significance codes: * = 0.05; ⋆ = 0.1

Coefficient	Estimate	Significance level
JGit		
*Error Term (Intercept)*	− 0.011	0.951
*Lines added*	− 3.42 ⋅ 10^− 4^	0.730
*Lines deleted*	2.97 ⋅ 10^− 3^	0.326
*N. files*	0.09	0.078 ⋆
*Size*	3.51 ⋅ 10^− 4^	0.012 *
Java-client		
*Error Term (Intercept)*	2.79 ⋅ 10^− 1^	0.774
*Author*		
SB	− 1.97	0.093 ⋆
MN	− 1.04	0.299
...		
*Lines deleted*	9.14 ⋅ 10^− 4^	0.475
*N. files*	− 5.88 ⋅ 10^− 2^	0.119
Android		
*Error Term (Intercept)*	− 2.74 ⋅ 10^− 1^	0.087 ⋆
*Lines deleted*	4.55 ⋅ 10^− 4^	0.269
*N. files*	− 2.55 ⋅ 10^− 2^	0.071 ⋆
*Size*	3.52 ⋅ 10^− 6^	0.551
Total		
*Error Term (Intercept)*	− 1.99 ⋅ 10^1^	0.998
*Author*		
AH	3.95 ⋅ 10^1^	0.998
BL	2.27 ⋅ 10^1^	0.998
...		
*Lines added*	− 6.44 ⋅ 10^− 4^	0.146
*Lines deleted*	4.03 ⋅ 10^− 4^	0.685
*N. files*	− 1.16 ⋅ 10^− 2^	0.580
*Size*	3.48 ⋅ 10^− 5^	0.254

The results obtained by our model for the four configurations are reported in Table 9. For JGit, the size of the files contained in the change-set under review is moderately correlated with the ratio of documented and undocumented changes. In the cases of Java-client and Android, the independent variables included in our models are not significantly correlated with our dependent variables, ratio of documented/undocumented review changes. This suggests that the author of a review change-set has no influence on the amount of triggered by comment changes in a review. Similarly the lines deleted do not seem to be correlated with a higher ratio of triggered by comment changes over undocumented changes in a revision. Overall, these findings give us an initial indication of what factors might be disregarded in further investigations on the cause of review changes. However, we cannot exclude that the lack of statistical significance is caused by an insufficient number of samples in our dataset. For this reason, further studies can expand our dataset and further explore this aspect of the code review process.

### RQ3.1—Factors Related to the Number of Review Changes

Our goal is to create four GLMs: three for each project (JGit, Java-client and Android) and one for the dataset that includes all three projects. As a first step in building our models, we evaluated the multi-collinearity across the considered variables. We removed the variable code churn from all modes since it was linearly dependent on other two metrics: lines added and lines deleted. After removing this variable, our analysis showed that all other variables had a (*GV* *IF*^(1/(2∗*Df*))^)^2^ value lower than 5. The only exception was the model for JGit, where the variable number of files achieved a (*GV* *IF*^(1/(2∗*Df*))^)^2^ = 15.7. After removing it from the model, we performed a further analysis confirming that no other variable had correlation value above 5. We built the models as follows:

**JGit.**: We included the following variables in the model: (1) Author, (2) lines added, (3) lines deleted, (4) size, (5) complexity, and (6) number of reviewers. Therefore, we constructed a GLM model using all the remaining variables. Our GLM achieved a *𝜃* = 0.3269 and a standard error of 0.0103. We reported the standard error instead of *R*^2^, following the advice given by Beller et al. (2014).
**Java-client.**: We included the following variables in the model: (1) Author, (2) lines added, (3) lines deleted, (4) size, (5) complexity, (6) number of reviewers, and (7) number of files. This first model showed that lines deleted, author, and number of files did not have a statistically significant value in the model. To verify if these three variables are statistically insignificant predictors of the dependent variable “Number of changes”, we proceeded as follows: We performed a *Chi-square* test between the models with and without the variable lines deleted. We obtained a *Pr*(*χ*) = 0.5725, above the statistically significant level of 0.05, which confirmed that lines deleted is a statistically insignificant predictor and, therefore, can be safely removed from the model. Then, we performed the same statistical test between the new obtained model and the same model without the variable Author. This test obtained a *Pr*(*χ*) = 0.4469, allowing us to remove Author from the model. Finally, we applied the same procedure to the new model to verify if number of files was a significant predictor. This test resulted in a *Pr*(*χ*) = 0.3648, confirming that number of files can be safely removed from the model. Our final model, obtained removing lines deleted, author, and number of files as explanatory variables, achieved a *𝜃* = 0.1452 and a standard error = 0.0109.
**Android.**: We removed the variable number of files as it achieved a (*GV* *IF*^(1/(2∗*Df*))^)^2^ higher than 5. No further variables achieved a multi-collinearity value higher than the threshold. Therefore, we included the following variables in the model: (1) Author, (2) lines added, (3) lines deleted, (4) size, (5) complexity, and (6) number of reviewers. Our model showed that the contribution given by *lines deleted* variable was not significant (*p* − *value* = 0.9442). For this reason, we verified if *lined deleted* is a significant predictor of the dependent variable *number of changes*. To this aim, we performed a *Chi-square* test between two models with and without this variable, respectively. The test achieved a *Pr*(*Chi*) = 0.9483, which is higher than the statistical significance level of 0.05. We can, therefore, remove the variable *lines deleted* from our model. The final GLM achieved *𝜃* = 0.4031 and standard error = 0.0094.
**Total.**: We included the following variables in the model: (1) Author, (2) lines added, (3) lines deleted, (4) size, (5) complexity, (6) number of reviewers, and (7) number of files. Our model highlighted that lines deleted do not seem to offer a statistically significant contribution (*p* − *value* = 0.239). To verify if lines deleted is indeed a statistically insignificant predictor of number of changes, we performed a *Chi-square* test between two models with and without including this variable. We achieved a *Pr*(*Chi*) = 0.502, which is higher than the significance level of 0.05. This allowed us removing this variable from the model. Then, we noticed how also the variables complexity and number of files did not present a statistically significant correlation with the dependent variable. As done for lines deleted, we performed a *Chi-square* test between the models with and without these variables, achieving a *Pr*(*Chi*) = 0.807 and *Pr*(*Chi*) = 0.152, respectively. Therefore, we removed both variables from the model. This procedure led us to a GLM with *𝜃* = 0.34491 and standard error = 0.00620.

Table 10 reports the results obtained by the four models. Overall, the coefficient ‘lines added’ has a positive correlation with the number of changes in a review (the dependent variable). This confirms our hypothesis that the larger a review change-set is, the more likely it is that the review will contain a higher number of changes. A similar effect can also be observed for the coefficient ‘number of reviewers’. The variable ‘churn’ was instead excluded from all our models since it was linearly dependent on ‘lines added’. Therefore, we can not draw any conclusion on our hypothesis that a larger churn leads to a higher number of comments.

**Table 10:** GLM models for the three projects separately and the complete dataset. Significance codes: *** = 0.001; ** = 0.01; * = 0.05; ⋆ = 0.1

Coefficient	Estimate	Significance level
JGit		
*Error Term (Intercept)*	1.10	0.0023 **
*Author*		
SK	− 1.44	0.0005 ***
TW	− 1.28	0.013 **
...		
*Lines added*	4.93 ⋅ 10^− 3^	< 2 ⋅ 10^− 16^ ***
*Lines deleted*	− 1.44 ⋅ 10^− 3^	0.0008 ***
*Size*	− 1.34 ⋅ 10^− 4^	< 2 ⋅ 10^− 16^ ***
*Complexity*	− 1.34 ⋅ 10^− 4^	2.11 ⋅ 10^− 9^ ***
*N. Reviewers*	4.01 ⋅ 10^− 1^	< 2 ⋅ 10^− 16^ ***
Java-client		
*Error Term (Intercept)*	7.22 ⋅ 10^− 1^	0.0063 **
*Lines added*	3.48 ⋅ 10^− 3^	1 ⋅ 10^− 13^ ***
*Size*	2.03 ⋅ 10^− 4^	0.0002 ***
*Complexity*	− 7.54 ⋅ 10^− 3^	0.0215 *
*N. Reviewers*	1.82 ⋅ 10^− 1^	0.0303 **
Android		
*Error Term (Intercept)*	7.35 ⋅ 10^− 1^	0.185
*Author*		
CS	3.62	6.69 ⋅ 10^− 4^ ***
CC	− 3.90	0.0012 **
...		
*Lines added*	2.18 ⋅ 10^− 3^	< 2 ⋅ 10^− 16^ ***
*Size*	5.92 ⋅ 10^− 5^	< 2 ⋅ 10^− 16^ ***
*Complexity*	3.02 ⋅ 10^− 3^	< 2 ⋅ 10^− 16^ ***
*N. Reviewers*	1.56 ⋅ 10^− 1^	< 2 ⋅ 10^− 16^ ***
Total		
*Error Term (Intercept)*	1.69	3.60 ⋅ 10^− 8^ ***
*Author*		
AP	− 2.50	5.09 ⋅ 10^− 9^ ***
CC	− 2.23	4.96 ⋅ 10^− 5^ ***
...		
*Lines added*	2.32 ⋅ 10^− 3^	< 2 ⋅ 10^− 16^ ***
*Size*	5.46 ⋅ 10^− 5^	< 2 ⋅ 10^− 16^ ***
*N. Reviewers*	2.48 ⋅ 10^− 1^	< 2 ⋅ 10^− 16^ ***

On the contrary, the variables ‘lines deleted’ and ‘complexity’ show an overall negative correlation with the dependent variable. Our findings seem to suggest that review change-sets containing less deleted lines are more likely to undergo a higher number of changes. Moreover, we cannot exclude that this result is linked to specific project considered as ‘lines added’ was not a significant predictor in the other models. Similarly, the complexity of a review change-set seems to be negatively correlated with the number of changes, with the exception of Android and the total model. This suggests that more complex reviews are slightly more likely to contain less changes. However, it is important to underline how this coefficient estimates is relatively small.

To gain further insight into which factors might impact the number of changes in a review, we computed the correlation among each independent variable and the number of review changes. As first step, we applied the Shapiro-Wilk test to check if the variables are approximately normally distributed. However, this test can not correctly handle datasets with more than 5000 instances. To avoid this limitation, for the dataset including all three projects, we resorted to the application of the Jarque-Bera test of normality.

These tests rejected the hypothesis that the data are normally distributed. Because of the not-normal distribution of the variables and the presence of ties, we applied Kendall correlation analysis. Table 11 shows the correlation coefficients computed in this way. These results allow us to compare the different metrics and identify the ones that have a higher contribution in predicting the number of changes in a review. Metrics like *lines added* and *complexity* show the highest, albeit still low, correlation with the number of changes in a review. On the contrary, the *number of reviewers* and *lines deleted* have the lowest correlation with the dependent variable among the considered metrics. Overall, the contribution of each metric is stable across the different projects.

**Table 11:** Correlation between explanatory and dependent variable (number of changes) for JGit, Java-client, Android and the whole dataset

Churn	N. files	Lines added	Lines deleted	Complexity	Size	N. reviewers
JGit						
0.279	0.232	0.302	0.102	0.238	0.213	0.275
Java-client						
0.263	0.216	0.275	0.071	0.198	0.164	0.191
Android						
0.307	0.211	0.338	0.145	0.274	0.130	0.112
Total						
0.284	0.209	0.306	0.127	0.243	0.156	0.174

Our analysis revealed how even metrics likely to have a high impact on the number of change have instead a surprisingly low correlation with the dependent variable. For instance, we expected metrics such as *churn* and *number of files* to be strongly correlated with the number of review changes, as a higher number of lines or files modified is more likely to have a higher number of changes. However, these metrics achieved only a small correlation (between 0.208 and 0.307) with the dependent variable. Similarly, the number of reviewers has an unexpected low correlation with the number of changes, ranging from 0.275 in the JGit project to 0.112 in Android.

### RQ3.2—Beyond only *merged* Reviews

As previously done in RQ3.1, we built four different Generalized Linear Models (GLMs), one for each project and a total considering all projects together. The following illustrates the creation and results of our models. The variable churn was removed in all models as it is aliased with the variable lines deleted.

**JGit.**: We removed the variable author from the model as it prevented the model from converging. Then, we checked the multicollinearity among the remaining variables and excluded size and number of files as they both presented a (*GV* *IF*^(1/(2∗*Df*))^)^2^ value higher than 5 (7.34 and 18.62, respectively). Then, we noticed that the variables lines deleted, status, reviewers experience, description length, and subject length did not seem to be statistically significant predictors of the dependent variables. Performing a *Chi-square* test between the original model and the models without these variables, we verified that we could safely remove all variables except from reviewers experience. Our final model, reported in Table 12 achieved a *𝜃* of 0.276 and a standard error of 0.008.
**Egit.**: We removed the variable topic as it was aliased with the variable branch. Then, we removed from the model the variable number of files as it presented a (*GV* *IF*^(1/(2∗*Df*))^)^2^ value of 7.779 (higher than the selected threshold of five). Finally, we noticed that the variables size, number of directories, subject length, number of bots, branch and status were not statistically significant predictors of the dependent variable. To verify if it was possible to remove them from the model, we performed, a *Chi-square* test, achieving a value higher than 0.05. Therefore, we removed these variables from the model. Our final model is reported in Table 12. The model achieved a *𝜃* of 0.228 and a standard error of 0.009.
**Platform UI.**: We removed the variable topic as it was aliased with branch. Then, we tested the collinearity of the remaining variables removing lines added, complexity, and number of files as they presented a (*GV* *IF*^(1/(2∗*Df*))^)^2^ higher than five. Finally, we performed a *Chi-square* test to evaluate if it was possible to safely remove variables with non-statistically significant contributions to the model. This test allowed us to remove the variables number of directories, number of bots, and subject length. However, we could not remove the variables author and reviewers experience as we achieved a *Pr*(*Chi*) lower than 0.05. The final model, which achieved a *𝜃* of 0.175 and a standard error of 0.005, is presented in Table 13.
**Total.**: We excluded the variables complexity, lines added, and number of files from the model has they presented a (*GV* *IF*^(1/(2∗*Df*))^)^2^ value higher than five. Then, we noticed that the variables number of directories and subject length were not statistically significant. After performing a *Chi-square* test between the models with and without these variables, we concluded it was possible to safely remove them from the model. Morever, we confirmed it was not possible to remove the variables status and reviewers’ experience from the model. Our final model is reported in Table 13.

**Table 12:** GLM models for the three projects separately and the complete dataset. Significance codes: *** = 0.001; ** = 0.01; * = 0.05; ⋆ = 0.1

Coefficient	Estimate	Significance level
JGit		
*Error Term (Intercept)*	9.53 ⋅ 10^− 1^	0.1425
*Lines added*	4.12 ⋅ 10^− 3^	< 2 ⋅ 10^− 16^ ***
*Complexity*	5 ⋅ 10^− 3^	4.03 ⋅ 10^− 8^ ***
*N. Reviewers*	4.95 ⋅ 10^− 1^	< 2 ⋅ 10^− 16^ ***
*Topic*		
notes	− 3.97	5.07 ⋅ 10^− 6^ ***
reftable	2.87	4.41 ⋅ 10^− 10^**
...		
*Branch*		
stable-4.2	− 3.23	0.0040 **
stable-3.7	− 3.23	0.0205 *
...		
*Author exp.*	− 1.85 ⋅ 10^− 3^	< 2.56 ⋅ 10^− 16^ ***
*Av. reviewers exp.*	− 9.34 ⋅ 10^− 3^	0.4261
*N. directories*	6.099 ⋅ 10^− 2^	0.0007 ***
EGit		
*Error Term (Intercept)*	− 2.42	0.1249
*Author*		
MH	4.77	0.0093 **
LG	4.08	0.0122 *
...		
*Lines added*	5.18 ⋅ 10^− 3^	< 2 ⋅ 10^− 16^ ***
*Lines deleted*	− 4.65 ⋅ 10^− 3^	5.37 ⋅ 10^− 6^ ***
*Complexity*	3.41 ⋅ 10^− 2^	2 ⋅ 10^− 16^ ***
*N. Reviewers*	5.92 ⋅ 10^− 1^	2 ⋅ 10^− 16^ ***
*Author exp.*	− 1.12 ⋅ 10^− 3^	0.0224 *
*Av. Reviewers exp.*	− 1.94 ⋅ 10^− 4^	0.0101 *
*Desc. length*	4.37 ⋅ 10^− 4^	0.0457 *

**Table 13:** GLM models for the three projects separately and the complete dataset. Significance codes: *** = 0.001; ** = 0.01; * = 0.05; ⋆ = 0.1

Coefficient	Estimate	Significance level
Platform UI		
*Error Term (Intercept)*	9.85 ⋅ 10^− 1^	0.7148
*Author*		
AY	− 4.89	0.0832 ⋆
DZ	3.28	0.2489
...		
*Lines deleted*	9.64 ⋅ 10^− 4^	< 2 ⋅ 10^− 16^ ***
*Size*	6.34 ⋅ 10^− 5^	< 2 ⋅ 10^− 16^ ***
*N. Reviewers*	6.85 ⋅ 10^− 1^	< 2 ⋅ 10^− 16^ ***
*Status*		
Merged	− 7.79 ⋅ 10^− 1^	< 3.59 ⋅ 10^− 7^ ***
New	6.32 ⋅ 10^− 1^	0.0807 ⋆
*Branch*		
R4_3_maintenance	− 4.31	0.0008 ***
R4_7_maintenance	− 4.25	0.0030 **
...		
*Author exp*	− 1.04 ⋅ 10^− 3^	< 2.86 ⋅ 10^− 16^ ***
*Av. Reviewers exp.*	− 4.57 ⋅ 10^− 5^	0.2398
*Desc. length*	1.34 ⋅ 10^− 3^	0.0003 ***
Total		
*Error Term (Intercept)*	1.99 ⋅ 10^1^	0.528
*Author*		
MS	5.87 ⋅ 10^2^	2.38 ⋅ 10^− 26^ ***
CH	1.03 ⋅ 10^2^	2.82 ⋅ 10^− 4^ ***
...		
*Topic*		
reftree	1.10 ⋅ 10^2^	4.67 ⋅ 10^− 6^ ***
moving-to-request-2	5.71 ⋅ 10^1^	4.80 ⋅ 10^− 4^ ***
...		
*Lines deleted*	− 1.67 ⋅ 10^− 3^	0.0172 *
*Size*	1.01 ⋅ 10^− 3^	9.31 ⋅ 10^− 21^ ***
*N. Reviewers*	6.24	< 1.06 ⋅ 10^− 23^ ***
*Authors exp.*	− 6.69 ⋅ 10^− 3^	0.0022 **
*Av. reviewers exp.*	6.41 ⋅ 10^− 5^	0.827
*N. bots*	9.73	3.18 ⋅ 10^− 7^ ***
*Status*		
merged	− 2.01	0.287
...		
*Branch*		
stable-4.5	− 4.26 ⋅ 10^1^	0.0251 *
stable-5.1	− 3.54 ⋅ 10^1^	0.0295 *
...		
*Desc. length*	6.65 ⋅ 10^− 3^	4.56 ⋅ 10^− 3^ **

Overall, our investigation confirmed the importance of metrics already employed in our previous analysis (e.g., number of reviewers). Moreover, it confirmed our initial hypothesis on the variable author experience: An author with more experience contributes code that requires fewer changes in the review.

Similarly to RQ3.1, the variable ‘lines added’ is positively correlated with the number of review changes, confirming our hypothesis that revisions containing a larger amount of lines added are likely to be subjected to a higher number of changes. A higher number of lines deleted is positively correlated with a higher number of changes in the Platform UI project, but not in EGit and in the Total model. This seems to suggest that developers consider lines deleted differently in the review compared to lines added.

Our findings confirmed also what reported by Rigby and Storey (2011): a longer description of a review patch-set seems to attract more attention from the reviewers which, in turn, translates into a higher number of changes (as confirmed by the positive correlation that this variable has with our dependent variable in EGit and Platform UI). Moreover, the topic or the branch to which a review change-set is linked seem to have an effect on the number of review changes. However, this effect depends on the specific project considered. Finally, in the total model, we can observe how the number of bots has a positive correlation with the number of changes in a review. This seems to confirm our initial hypothesis that the presence of bots in a review makes a patch undergo a higher number of changes.

## Discussion

Overall, the results of **RQ1** confirm the trend already observed by previous studies (Mäntylä and Lassenius 2009; Beller et al. 2014; Siy and Votta 2001): Evolvability changes constitute the vast majority of the changes happening during code review, with a ratio of approximately 9:1 compared to functional changes.

Also at type level we corroborated previous findings, showing how the vast majority of changes belong either to the ‘documentation’ or ‘structure’ group. Among the functional changes, the majority of them belong to the ‘check’, ‘interface’, and ‘logic’ type. Overall, their distribution is similar to the one shown in previous studies (Mäntylä and Lassenius 2009; Beller et al. 2014). However, Beller et al. (2014) reported a percentage of logic changes approximately between 10% and 15%, depending on the system under analysis. In our analysis, ‘logic’ changes only counted for slightly less than the 5% of the changes. This difference might be caused by multiple factors, such as the different projects under analysis.

At sub-type level, the majority of changes belong to the “comments” sub-type, reflecting issues in the comments that have been fixed at code review time. Comparing this result with the findings of Mäntylä and Lassenius (2009), a similar amount of “comments” changes was reported in the students’ reviews, but a significantly lower number was reported in the industrial reviews. This difference might be caused by the presence of many newcomers or “casual” contributors in the open-source projects considered in our analysis. These developers might not have been familiar with the style guidelines of the project. On the other hand, developers in the company considered by Mäntylä and Lassenius (2009) worked full time on the project and might have been therefore, more familiar with its style and guidelines. Open-source might, therefore, benefit from more explicit guidelines on coding style that contributors are asked to read before taking part into the project. A similar reasoning might also apply to “visual representation” changes. In the considered open-source projects, the amount of kind of changes is considerably higher than in the industrial reviews considered by Mäntylä and Lassenius (2009).

Concerning organizational changes, the majority of them belong to the “dead code” and “move functionality”, confirming the findings by Mäntylä and Lassenius (2009). This result suggests that developers use code review not only to identify defects or make localized improvements to the code, but also to discuss the design of the system. However, organizational changes as a whole constitute only approximately 9% of the changes happening during code review.

The analysis conducted to answer **RQ2** revealed that the majority of the changes have *not* been triggered by a comment. This highlights a different trend from the one reported by Beller et al. (2014), where on average only 15% of the changes were undocumented. This discrepancy in the results can have multiple causes, all having roots in the difference between the project considered in these two studies. Different code review policies might have been applied to these projects. This could also explain the internal difference in our dataset between the first two systems under analysis (Android and JGit) and Java-client. Furthermore, we noticed the presence of many self-triggered changes, often happening when the author of a patch uploads further patches to make follow-up changes without involving reviewers. We hypothesize that this practice might be a possible cause of the discrepancy we noticed. Another possible hypothesis is that the low number of changes triggered by comments might have been caused by the reviewers discussing the changes in places other than Gerrit (e.g., developers’ mailing list). Further studies should be devised to investigate the causes behind this low number of “triggered by comment” changes.

Our analysis did not find any statistically significant correlation between the cause that triggered a change and its category. While conducting our investigation, we only noticed a weak correlation between the cause of a change and its type for the dataset containing the three projects. Our hypothesis was that particular kinds of changes (e.g., functional changes) were more likely to be caused by a reviewers’ comment. However, our results prevented us from drawing any strong conclusions.

Our investigation focused on the use of Gerrit by the developers. Therefore, we can not exclude the possibility that changes were discussed on other platforms. For this reason, future studies should explore other communication platforms of the developers to collect further insight into undocumented changes. Moreover, future research might also focus on devising qualitative studies (employing, for instance, interviews with developers) to further extend our findings.

The analysis of the GLMs created in **RQ3** showed that lines added, size, and number of reviewers are statistically significant factors in predicting the number of changes in a review. This holds true for the models created for a single project as well as the model taking into account the whole dataset. This finding confirms the goodness of our model. Furthermore, it corroborates previous findings in the literature (Beller et al. 2014) that reported how the dimension of a change (expressed as size or lines added) has an impact on the amount of changes that will happen in the code. Moreover, a higher number of reviewers likely leads to a higher number of comments or review rounds and, therefore, to a higher number of changes. However, when considered independently these metrics show a relatively low level of correlation with the dependent variable (number of changes). For instance, the number of reviewers has only a between low level of correlation (ranging from 0.112 to 0.275 depending on the project). These findings suggest that none of the considered metrics is sufficient alone, but they can achieve good results when combined in a unique model.

### Implications for Researchers and Practitioners

In this study, we provide further evidence on the outcome of code review. We found, for example, that the majority of the changes in our dataset are low-level maintainability defect and not severe functional defects. The kind of data we produced is fundamental for the creation of *code review analytics* tool to assess the real outcomes of this process. For instance, researchers at Microsoft developed *CodeFlow Analytics* (CFA), an analytics tool created to collect and display information about the code review process (Bird et al. 2015). The request for such a tool came from developers teams, interested in a way to monitor their code reviews. Moreover, the use of CFA led to two fundamental outcomes: (1) developers were able to monitor and improve their processes and (2) fostered opportunity for further research to improve the code review process. Given the benefits achieved by CFA, we believe future research should focus on the creation of similar code review analytics tools. To this aim, information on the kind of review changes might prove fundamental to guide researchers’ efforts: e.g., by identifying the most common kinds of review changes and developing approaches to automatically classify them.

Although Gerrit is the ideal candidate to extract information to be used by code review analytics tools (as it was also confirmed by our investigation), the high number of undocumented changes contained in our dataset seems to suggest that Gerrit alone is not enough: To obtain information on developers’ information exchange during code review, future code review analytics tools should combine the information available on Gerrit with other sources of information (e.g., developers’ mailing lists).

Although the findings of RQ1 are more directly linked to the creation of review analytics tools, also the results of RQ2 and RQ3 can be informative for this purpose. For instance, the findings of RQ3 can suggest relevant metrics to include in code review analytics: e.g., the branch or the description length of the review patch-set, as these metrics are correlated with the complexity of the review.

Our investigation of possible factors that might influence the number of changes in a review might lead the way towards the creation of *review prioritization* systems: Tools to rank and flag open reviews based on their complexity, allowing reviewers and managers to better plan and allocate their resources. To this aim, understanding which metrics of a review might be good predictors of its complexity is paramount. Our investigation contributed to this direction by investigating an initial set of metrics and collecting insights that might inform future studies in the field. For instance, the number of lines deleted in the review change-set does not seem to be correlated with the number of changes (with the exception of JGit) and, therefore, could be disregarded in future investigation. Although our findings do not lead to “production-ready” solutions, we believe they can be beneficial to guide further research on task prioritization. Moreover, even without the support of automated task prioritization tools, practitioners can leverage our findings to perform initial estimations of the effort required to review a PR.

Another possible research direction is to consider if review factors are correlated with the presence of specific code smells. Further research could exploit our dataset and link it to the analysis of code smells in the reviews contained in it.

## Threats to Validity

### Internal Validity

To generate the dataset used in our analysis, we manually labeled each change assigning a category, type, and subtype. Furthermore, we also manually determined the cause of a change. This labeling process might have introduced bias in the dataset, as the authors were not directly involved in the selected projects (e.g., having contributed to them). To mitigate this, we computed the inter-rater agreement between the first two authors on a statistically significant subset of changes, computed with a confidence level of 95*%* and margin of error of 5*%*, achieving an inter-rater agreement of 90.52% at category level, 73.87% at type level, and 64.7% at subtype level in our RQ1, and an inter-rater agreement of 91.11% in RQ2.

### External Validity

In our analysis, we considered three projects: Android, JGit and Java-client. Although these have been often used to investigate the code review process in Open-Source environments due to their characteristics, we can not discard the possibility that our selection of projects introduced bias in the consequent analysis. Moreover, in our investigation we focused on *merged* reviews to obtain a picture of the successful code review practices of the project. However, we can not exclude that this choice might have introduced bias in our dataset. To investigate the impact of this choice in our RQ3 (Section 4.4) we also included the *status* (e.g., merged) of a review in the models to evaluate if this has a statistically significant impact on the number of changes in a review.

Analyzing JGit and Java-client, we limited our investigation only to Java files. This choice was taken to increase the reliability of the results of our manual classification because of the authors’ expertise with the Java language. However, to mitigate the possible bias introduced by this choice, in Android we analyzed all changes included in a review change-set, regardless of their programming language. Moreover, the results of **RQ1** are in-line with the findings of Beller et al. (2014), where the authors focused on GROMACS, a system mostly written in C. Nonetheless, we can not exclude our projects selection might have introduced bias in the dataset (for instance because of different review practices linked to specific programming languages). For this reason, further studies might be necessary to evaluate if these findings remain valid also for other programming languages: e.g., Python.

All projects considered to create our dataset use Gerrit as code review tool. Gerrit is a popular code review tool used my many open-source projects (including projects like Android or QT that have been shown to be highly representative of the open-source review process) (Pascarella et al. 2018; McIntosh et al. 2014; Bavota and Russo 2015). Even though code review tools offer very similar capabilities (Baum and Schneider 2016), we can not exclude that the choice of a different code review tool (e.g., GitHub) might have led to different results.

We included in our dataset only changes extracted from reviews successfully merged into the codebase of the project. Although our focus was to investigate only the factors characterizing succesful reviews, this choice might limit the generalizability of our findings to the whole code review process of a project containing also unmerged and in-progress reviews.

In **RQ2**, we focused only on reviews leading to subsequent changes to investigate the ratio between changes triggered by a comment over the whole number of comments in a revision. However, the features used in our model might not be unique only to change-inducing reviews. Further studies can be devised and conducted to compare the features of change-inducing reviews to those of reviews that did not lead to any change in the code.

In **RQ3**, we limited our selection of reviews to those containing only java files. This decision might have introduced bias in the results of GLM compared to a GLM created for the whole review corpus. The value computed for some variables used in our model, for example *Complexity*, might be different when considering different programming languages. It is possible that reviewers are added to the review during the process. This might have introduced bias in the computation of the variable number of reviewers since the computation is not able to take into account this kind of situations.

## Conclusion

In this study, we aimed to answer three research questions to deepen our scientific understanding of review changes.

We classified the changes based on their category (evolvability or functional), type and sub-type. To do so, we referred to previously created taxonomies (Beller et al. 2014; Mäntylä and Lassenius 2009). Our findings confirmed the trend reported by previous work: The vast majority of changes happening during the code review process are related to evolvability issues. At type level, we reported a prevalence of documentation and structure changes.

Then, we investigated what caused a change: a reviewers’ comment or another form of interaction not registered in Gerrit (undocumented change). Our investigation revealed that for the changes contained in our dataset, the vast majority belong to the latter class. Furthermore, we tested possible correlation among the cause of a change and its category and type. Unfortunately, this approach found only a weak association between the cause and type of a change considering the whole dataset formed by the three projects.

Finally, we analyzed which factors can have an impact on the number of changes that happened in a review. To this aim, we created four Generalized Linear Models (GLMs), one per project and one for the whole dataset. These models showed promising results and confirmed some of our hypotheses: in particular, it showed that the size of the code under review (in terms of total number of lines and lines added) is a significant factor to predict the number of changes.

## Data Availability

The code developed in the context of this study is available in our replication package at the following link: 10.5281/zenodo.6811165.

## Footnotes

1 Online taxonomy: https://figshare.com/s/7cdb9faa37a6e869b2e1

2 Replication package: 10.5281/zenodo.6811165.

3 gerrit-rest-java-client: https://github.com/uwolfer/gerrit-rest-java-client

4 Using the following online tool: http://dfreelon.org/utils/recalfront/recal2/

5 java-gerrit-rest-api: https://github.com/uwolfer/gerrit-rest-java-client

6 Replication package: 10.5281/zenodo.6811165

7 Online taxonomy: https://figshare.com/s/7cdb9faa37a6e869b2e1
